# Sun protection behavior beliefs among adults living in rural United States: A qualitative study in Minnesota

**DOI:** 10.1371/journal.pone.0331685

**Published:** 2025-09-12

**Authors:** Patricia Jewett, Matia Solomon, Katherine Brown, Deveny Flanagan, Emma Kelly, Kelly Kunkel, Megan Schossow, Zora Radosevich, Pamela Mason, Rehana Ahmed, Rebekah Nagler, Carrie Henning-Smith, DeAnn Lazovich, Marco Yzer, Rachel I. Vogel

**Affiliations:** 1 Division of Environmental Health Sciences, University of Minnesota, Minneapolis, Minnesota, United States of America; 2 Department of Obstetrics, Gynecology and Women’s Health, University of Minnesota, Minneapolis, Minnesota, United States of America; 3 University Survey & Assessment Services, University of Minnesota, Minneapolis, Minnesota, United States of America; 4 University of Minnesota Extension, Mankato, Minnesota, United States of America; 5 Upper Midwest Agricultural Safety and Health Center (UMASH), University of Minnesota, Minneapolis, Minnesota, United States of America; 6 Minnesota Department of Health, St. Paul, Minnesota, United States of America; 7 American Cancer Society, Eagan, Minnesota, United States of America; 8 Department of Dermatology, University of Minnesota, Minneapolis, Minnesota, United States of America; 9 Masonic Cancer Center, University of Minnesota, Minneapolis, Minnesota, United States of America; 10 Hubbard School of Journalism & Mass Communication, University of Minnesota, Minneapolis, Minnesota, United States of America; 11 Division of Health Policy and Management, University of Minnesota, Minneapolis, Minnesota, United States of America; 12 Division of Epidemiology and Community Health, University of Minnesota, Minneapolis, Minnesota, United States of America; The University of Sydney, AUSTRALIA

## Abstract

Although skin cancers are considered mostly preventable, more people in the US are diagnosed with skin cancer than all other cancers combined. Sun safety recommendations include wearing sun-protective clothing, a wide-brimmed hat, seeking shade, and using sunscreen. Some evidence exists that sun risk behaviors and skin cancer rates are more frequent among rural than urban US populations, raising questions about underlying factors. We conducted a belief elicitation survey on these four sun protection behaviors among 278 adults (aged 18–60 years) living in rural Minnesota, a state with high sunburn rates and UV-attributable melanoma cases. These qualitative data were analyzed using content analysis, and the identified codes ranked by frequency. Almost all participants emphasized that spending time outside was important to them. The most frequently reported sun protection behaviors were wearing sunscreen and protective clothing. The primary outcomes were obtained from open-ended questions on outcome, normative, and control beliefs associated with each sun protection behavior. While many different beliefs were mentioned, reducing sunburn and skin cancer risk were commonly reported across all behaviors. Beliefs about negative aspects of each behavior (e.g., interference with being physically active or doing work outside, greasy/sticky sunscreen, not getting a suntan, overheating in long clothes or when wearing hats, hats that blow off easily) typically outnumbered positive aspects (e.g., protective behaviors enabling being outside, staying cool in shade, reduced skin aging). The majority of participants believed that most people would approve of all protection behaviors, but many thought that age was a factor for behavior adoption, with young people typically thought to engage less in protective behaviors. Some commonly reported negative aspects of sun protective behaviors were related to activities more common in rural populations, such as working outside. This suggests that rural sun protection promotion may include structural interventions to make sun protection easy, convenient, and accessible without impeding rural lifestyles.

## Introduction

Skin cancer is increasing beyond what would be expected by increased screening alone [1,2], and more people are diagnosed with skin cancer each year in the United States (US) than all other cancers combined [3]. Skin cancers are largely preventable, with excessive ultraviolet radiation (UVR) exposure being one of the strongest risk factors [4–6]. Sunburn rates in the US have remained consistent over the last 30 years, with over one-third of adults reporting at least one sunburn in the last year [7]. Recent work suggests that skin cancers, including melanoma incidence [8], are more frequent in rural than in urban areas of the US.

Rural-urban differences in skin cancer patterns intersect with regional differences across the US [9]. Minnesota, with its location in the north-central US, is fourth in the country for UVR-attributable melanoma [10], and sunburn prevalence is higher in the Midwest than in the Northeast, South, or West regions of the US [11]. This is likely due in part to weather-related intermittent sun exposure characteristic of northern climates and differences in rural versus urban lifestyle and sun behaviors. More Minnesotans (23%) live in non-metropolitan counties compared to the US overall (15%) [12]. They are more likely to be employed in agriculture jobs compared to most other states and to live in poverty [13], social factors which may be related to sun risk and protection behaviors.

To prevent sunburns and skin cancer, the Centers for Disease Control and Prevention (CDC) recommends wearing sun-protective clothing, sunglasses, and a wide-brimmed hat; seeking shade; avoiding the sun between 10am and 4 pm; and using sunscreen with a sun protection factor (SPF) of 15 or greater [14]. More risky sun behaviors (infrequent sunscreen and protective clothing use, not seeking shade) and potentially greater exposure from increased time spent outside are more often reported in rural than urban populations [15–19], and rural residents with skin cancer more often present with advanced stage disease [20]. Risk behaviors, barriers to care, and financial hardship make skin cancer prevention paramount in rural areas [21].

UVR exposure can be classified as intentional (purposely exposing one’s skin to tan) versus incidental (exposure as a byproduct of occupational or recreational activities). Data suggest that most sunburns occur during incidental exposures—for example, working outside around the house or when exercising outdoors—and not when tanning intentionally [22–25]. Individuals living in rural versus urban areas often engage in different outdoor activities and these unique patterns may influence skin cancer risk. For example, a study in the Western region of the US found that the main outdoor activities associated with metropolitan families included going to amusement parks, outdoor exercise, water activities, and outdoor play, while the main outdoor activities engaged in by rural families were farm work, sports, yard work, and other outdoor chores [26].

In order to understand rural sun behaviors, it is important to understand underlying beliefs and attitudes. Authoritative behavioral theories such as the reasoned action approach [27] have been prominently used in many health domains, and have been successful in explaining determinants of sun-protective behaviors [28]. The reasoned action approach explains that behavior is ultimately guided by beliefs about outcomes of engaging in the particular behavior, beliefs about normative pressure for or against the behavior from specific referents, and beliefs about control-related factors that facilitate or hamper performing the behavior.

*Which* beliefs influence behavior are unique to every population and for every behavior. Therefore, the first critical step is to understand beliefs in the population of interest. Once beliefs are understood, they can inform tailored public health messaging based on the idea that successfully addressing these beliefs can change behaviors downstream [29–31]. Beliefs and related perceptions about skin cancer have been identified in the general population: self-reported barriers to reducing sun exposure include sunscreen feeling uncomfortable, forgetting to put on sunscreen, and not getting tanned [32–36]. However, it is unknown what skin cancer beliefs are most pertinent in rural populations.

Since successful behavior change requires both a behavior- and population-specific focus, it is important to identify pertinent beliefs, including misconceptions and inaccurate beliefs, and relevant structural barriers in rural communities. Therefore, we conducted a qualitative study with the aim to collect rural-specific outcome, normative, and control beliefs that underlie sun protection behaviors in adults living in rural Minnesota. The goal of this qualitative analysis was to inform survey development for future in-depth work to examine the frequencies of these beliefs relevant to rural sun behaviors.

## Methods

We conducted a qualitative belief elicitation survey focused on four sun protection behaviors for skin cancer prevention: wearing sun-protective clothing, wearing a wide-brimmed hat, seeking shade, and using SPF 15 + sunscreen. To complete this work, we assembled a team of researchers and staff members from community partners focused on improving sun behaviors and rural health. We collaborated with community partners from study inception to ensure relevance of the study questions and to support dissemination of study results. The study was deemed exempt and approved by the University of Minnesota Institutional Review Board (STUDY00014346), therefore documentation of written consent was waived. Implicit consent was considered if people completed the study survey. Participants submitted the survey data anonymously.

### Study design

We designed a belief elicitation survey based on theory recommendations [27] and design and analysis recommendations [37]. A belief elicitation survey uses open-ended questions with the goal to identify outcome, normative, and control beliefs associated with, in the present research, four sun protection behaviors among individuals living in rural areas in the United States. Belief elicitation methods use open-ended questions in order to avoid prompting participants to think in any particular direction; rather, open-ended questions measure top-of-mind beliefs, which is consistent with our aim to identify which beliefs individuals living in rural areas hold about the four sun protection behaviors [38,39]. To reduce participant burden, each participant was only asked to respond regarding one or two behaviors rather than all four. We aimed for 20–30 participants from each of the four age (18–39 years old, 40–60 years old) and gender strata because we anticipated potential gender and age differences.

### Study population

The targeted study population was individuals 18–60 years old living in rural Minnesota (Rural–Urban Commuting Area (RUCA) codes 4–10). We focused on young to middle-aged adults (ages 18–60 years) because they have significant life years remaining to prevent skin cancer. Sunburns rates are relatively consistent between ages 18 and 49 (~40% report at least one sunburn in the past 12 months) with a drop for those 50–59 years old (32%) and then a significant drop after 60 years old (<20%) [7].

### Sampling design

We collaborated with the Center for Survey Research in the Office of Measurement Services (OMS) at the University of Minnesota for recruitment and survey data collection. The address-based participant sample was purchased from Dynata, LLC of Shelton, Connecticut. First the sample was restricted to rural census tracts in Minnesota. Next, the sample was stratified equally by two age categories and two gender categories (Women 18–39, Women 40–60, Men 18–39, Men 40–60) to balance responses from these cells. Addresses that included individuals who satisfied these constraints were then randomly selected.

Two waves of survey distribution were completed. The response totals for Wave 1 did not meet necessary quotas so a second wave was also completed, shortening the survey to include only one behavior to reduce respondent burden. Wave 1 consisted of 1,200 addresses that were stratified such that an even number of addresses in the four respondent categories received each version of the survey. For Wave 2, the sample consisted of 1,000 addresses. Because the sample from Wave 1 had a higher number of completed surveys from older women, the Wave 2 sample was weighted to make up the differences.

### Recruitment

Recruitment occurred between June 10, 2022 and January 6, 2023. We followed the Dillman method of recruitment to optimize responses rates [40], which included an initial mailing (cover letter, survey, and a stamped return envelope), a postcard reminder one week later, a second mailing (another cover letter, paper survey, and stamped return envelope) sent to all non-completers, and a final reminder letter with a link to the online survey. Following this method, Wave 1 included a $2 bill in the first mailing for every household. Wave 2 utilized a different approach and provided a $10 cash incentive after submitting a completed survey. All documents were available in English only. Participants were assigned a study ID to ensure returned surveys were anonymous but trackable.

### Data collection and measures

**Fig 1:**
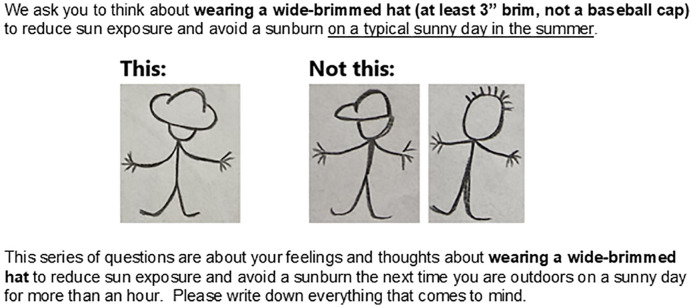
Example description and cartoon drawing of wearing a wide-brimmed hat to reduce sun exposure included in survey.

Respondents were able to complete and return a mailed paper survey, or follow a URL or QR code to take an online survey via REDCap [41]. Following established procedures [27,42], the survey described the sun protection behavior of interest using text and cartoon drawings (example Fig 1) and then asked a number of open-ended questions about that specific sun protection behavior. The open-ended questions asked participants to write down what they think the positive and negative consequences of using the specific sun protection method are (i.e., outcome beliefs), who they believe will approve or disapprove the behavior (i.e., normative beliefs), and which circumstances they believe might facilitate or hamper their use of the sun protection method (i.e., control beliefs; see Table 1 for example).

**Table 1:** Assessing outcome, normative, and control beliefs about wearing a wide brimmed hat.

Belief type	
**Outcome beliefs**	What do you see as the pros or benefits if you would **wear a wide-brimmed hat** the next time you are outdoors on a sunny day for more than an hour?
	What good or positive things come to mind when you think about if you would **wear a wide-brimmed hat** the next time you are outdoors on a sunny day for more than an hour?
	What do you see as the cons or downsides if you would **wear a wide-brimmed hat** the next time you are outdoors on a sunny day for more than an hour?
	What bad or negative things come to mind when you think about if you would **wear a wide-brimmed hat** the next time you are outdoors on a sunny day for more than an hour?
**Control beliefs**	What factors or circumstances would make it easier for you to **wear a wide-brimmed hat** the next time you are outdoors on a sunny day for more than an hour?
	What factors or circumstances would make it more difficult for you to **wear a wide-brimmed hat** the next time you are outdoors on a sunny day for more than an hour?
**Normative beliefs**	Which people or groups do you think would approve of you **wearing a wide-brimmed hat** when you are outdoors on a sunny day for more than an hour?
	Which people or groups do you think would not approve of you **wearing a wide-brimmed hat** when you are outdoors on a sunny day for more than an hour?
	Which people or groups do you think are most likely to **wearing a wide-brimmed hat** when they are outdoors on a sunny day for more than an hour?
	Which people or groups do you think are unlikely to **wearing a wide-brimmed hat** when they are outdoors on a sunny day for more than an hour?

To reduce participant burden, each participant was asked to complete the survey for 1 or 2 randomly assigned behaviors. For Wave 1, we asked each participant about two behaviors, split as follows: sunscreen and hat or shade and clothing, with the behaviors randomly ordered. For Wave 2, each participant was randomly asked about one behavior to further shorten the length of the survey to encourage completion.

The survey concluded by asking closed-ended questions regarding importance of spending time outside, typical sun behaviors [43], and demographics including age, gender, race, education, employment, marital status, and annual household income.

### Data analysis

We conducted a content analysis of the open-ended responses. Consistent with belief elicitation procedural and analysis recommendations for qualitative data [27,37,44], the responses were first read independently by two investigators (RIV and PJ) to identify beliefs for each question for each behavior and create a codebook. After the codebook was agreed upon, two raters (MS, KB) then independently re-read all participant responses and identified which beliefs were present in each answer to each question, following the codebook. Where they identified differing codes from those in the codebook, the new codes were reviewed and either rectified or added by a third rater (PJ). The codes were then compiled into themes and summarized based on the frequency of their report, overall and by age/gender strata. Participant demographic characteristics were summarized using descriptive statistics. This manual qualitative data analysis approach is based on Middlestadt (2012), who provides a detailed description of content-analyzing belief elicitation responses about multiple behaviors in multiple priority groups [37].

## Results

Of the 2,200 invitations sent, 418 were returned to sender, 3 were deceased, 19 refused, 1,439 did not return the survey, and 32 were either outside of the age range or did not provide their age; a total of 278 surveys from eligible participants were completed (12.6% of all sent; 15.6% of those reached). Most surveys were completed via paper (180; 64.7%), with the remaining completed online (98; 35.3%).

Demographic characteristics of the study sample are presented in Table 2. Study participants were relatively evenly distributed by age group and gender. Most participants were non-Hispanic White (94%), almost half had at least a college degree (47%), and nearly all were working (90%).

**Table 2:** Participant demographics (N = 278).

Characteristic	N	%
**Age group**		
18–39 years	132	47.5
40–60 years	146	52.5
**Gender**		
Woman	153	55.0
Man	125	45.0
**Race/ethnicity**		
Non-Hispanic White	250	94.0
American Indian/Alaska Native (AIAN)/AIAN + White	7	2.6
Hispanic, any race	4	1.5
Non-Hispanic, other race	5	1.9
Race or ethnicity missing	12	
**Education**		
High school degree or less	53	19.1
Some college/vocational/business school	95	34.3
At least college degree	129	46.6
Missing	1	
**Annual Household Income**		
< $50,000	58	22.4
$50,000-$99,999	99	38.2
$100,000 or more	102	39.4
Not reported	19	
**Marital status**		
Married/Partnered	180	65.2
Not partnered	96	34.8
Missing	2	
**Work status**		
Working part or full time	249	89.9
Retired	9	3.2
Not working	19	6.9
Missing	1	
**Outdoor job (among working)**		
No	191	77.3
Yes	56	22.7
Missing	2	
**Skin cancer personal history**		
No	256	92.1
Yes	17	6.1
Unsure	5	1.8

Almost all participants reported that it was at least moderately important to them to spend time outside in warm weather (95%) and over half (55%) spent two or more hours outside on weekdays between 10am and 4 pm, and over 90% spent two or more hours outside on weekend days between 10am and 4 pm (Table 3). Among the sun protection behaviors assessed, the most consistently reported sun protection behaviors on a sunny day were wearing sunscreen (39% always/often) and protective clothing (60% always/often). While these numbers were mostly congruent with participants’ willingness to engage in these behaviors (sunscreen 67%, protective clothing 55%), only 16% reported always or often wearing a wide brimmed hat although 60% reported being willing to do so.

**Table 3:** Self-reported sun exposure and protection behaviors (N = 278).

Characteristic	N	%
**Importance of spending time outside in warm weather**		
Extremely/very/moderately important	262	94.6
Slightly/not at all important	15	5.4
*Missing*	*1*	
**Weekdays, time outside 10am-4 pm**		
0-1h	124	44.9
2-3h	84	30.4
4h or more	68	24.6
*Missing*	*2*	
**Weekend days, time outside 10am-4 pm**		
0-1h	26	9.7
2-3h	76	28.5
4h or more	165	61.8
*Missing*	*11*	
**Number of sunburns in past 12 months**		
0	115	41.5
1	73	26.4
2 or more	89	32.1
*Missing*	*1*	
**Sunny day outside – wear sunscreen**		
Always/often	108	38.8
Sometimes	90	32.4
Rarely/never	80	28.8
**Sunny day outside – wear long sleeves**		
Always/often	166	60.1
Sometimes	64	23.2
Rarely/never	46	16.7
*Missing*	*2*	
**Sunny day outside – hat with brim**		
Always/often	43	15.6
Sometimes	54	19.6
Rarely/never	178	64.7
*Missing*	*3*	
**Sunny day outside – stay in shade**		
Always/often	56	20.2
Sometimes	124	44.8
Rarely/never	97	35.0
*Missing*	*1*	
**Sunny day outside – time in sun to tan**		
Always/often	46	16.5
Sometimes	68	24.5
Rarely/never	164	59.0
**Willing to wear sun protective clothing**		
Very/moderately willing	152	55.1
Slightly/not at all willing	124	44.9
*Missing*	*2*	
**Willing to wear sunscreen daily**		
Very/moderately willing	186	67.1
Slightly/not at all willing	91	32.9
*Missing*	*1*	
**Willing to wear hat**		
Very/moderately willing	167	60.3
Slightly/not at all willing	110	39.7
*Missing*	*1*	
**Willing to learn more about sun behaviors**		
Very/moderately willing	143	51.4
Slightly/not at all willing	135	48.6

### Overall

Commonly reported beliefs (provided by at least 10 individuals) about all sun protective behaviors (using sunscreen, staying in the shade, wearing protective clothing, wearing a wide-brimmed hat), are presented by overall frequency in Tables 4; all reported beliefs and detailed frequencies, stratified by age and gender, are provided as supplemental material (S1-S4 Tables). Most participants reported that reduced risk of sunburn and skin cancer was a positive aspect of engaging in each of the four sun protection behaviors. In total, we elicited 42 outcome beliefs about sunscreen use, 36 about staying in the shade, 45 about wearing protective clothing, and 30 about wearing a wide-brimmed hat. Only a small number of beliefs about each behavior were consistently shared across a substantial number of participants, with many beliefs expressed by only one or a few participants. We also summarized the number of negative and positive reported outcomes for each behavior stratified by gender and age group (S1–S4 Figs). Except for staying in the shade, the number of negative outcomes reported was higher than the number of positive reported outcomes. Women tended to report more positive outcomes of each behavior than men, and older individuals tended to report more positive outcomes than younger participants.

**Table 4:** Commonly reported beliefs about sun protection methods to reduce sun exposure and prevent sunburn on typical sunny day in the summer.

Using Sunscreen n = 111		Seeking Shade n = 96	
*Outcome*			
**Good/Positive** Less cancer/ exposure/sunburn Prevent skin aging/ less skin damage	**Bad/Negative** Greasy/sticky/ messy There is nothing is negative about sunscreen Time consuming/ difficult to apply Need to reapply Chemicals/ ingredients Inconvenient/ need to bring No suntan Gets in eyes Irritates skin/acne/ allergic reaction Expensive/cost Stains/leaves residue Sweating it off	**Good/Positive** Less cancer/ exposure/ sunburn Protection from heat/ stay cool Feel comfortable Be outside without sun exposure Prevent skin aging/ less skin damage	**Bad/Negative** There is nothing is negative about seeking shade No suntan/pale skin Away from others/ activity/social isolation Less vitamin D Bugs/Insects Too cool (miss warmth of sun) Lower work productivity/work not in the shade Not always available/need to plan
***Normative***			
**Approve/Support Use** Most people/ everyone Family/ friends Healthcare providers Parents/people with children	**Disapprove/Not Support Use** No one disapproves Young people	**Approve/Support Use** Family/ friends Most people/ everyone Healthcare providers Older people	**Disapprove/Not Support Use** No one disapproves Young people People who want to be tan/ in the sun Employers
**Likely to Use** People with fair skin/ prone to sunburn Parents/people with children Children Older people People with personal/family history of skin cancer	**Unlikely to Use** Young people Men People with dark skin People who are active outside (e.g., doing work/ workouts) Older people	**Likely to Use** Older people People with fair skin/ prone to sunburn People with personal/family history of skin cancer People who get hot/sweaty Parents/people with children	**Unlikely to Use** Young people People who want to be tan/ in the sun People who are active outside (e.g., doing work/ workouts)
***Control***			
**Facilitators/Easier to Use** Easily accessible Type of sunscreen available Easier to apply Not greasy/sticky Cheaper/ free No additional facilitators needed	**Barriers/Harder to Use** Not having nearby access or don’t own Forgetting No barriers Too busy/time consuming	**Facilitators/Easier to Use** Availability of shaded areas Availability of trees Canopy/pavilion at location Really hot/humid	**Barriers/Harder to Use** No shade available Job/work requires sun exposure No/not many trees No shade near activity Temperature too cool
Wearing Protective Clothing n = 117		Wearing wide-brimmed hat n = 105	
***Outcome***			
**Good/Positive** Less cancer/ exposure/sunburn Don’t have to apply sunscreen Less bug bites/less bug spray Protection from heat/ stay cool Reduce skin peeling/sunburn discomfort Prevent skin aging/ less skin damage	**Bad/Negative** Sweat/ overheating Uncomfortable/skin irritation No suntan/pale skin Cost/need to buy new clothes There is nothing is negative about protective clothing Tight/restricted movement during activity	**Good/Positive** Less cancer/ exposure/sunburn Sun out of eyes Protection from heat/ stay cool	**Bad/Negative** Blows off/easy to lose Head gets hot/sweaty Inconvenient/ interferes with work/task Not fashionable/ does not look good Uncomfortable/does not fit well There is nothing is negative about wearing a hat Hair not styled properly/ messes up hair Obstructed view
***Normative***			
**Approve/Support Use** Family/ friends Healthcare providers Older people Most people/ everyone People with personal/family history of skin cancer	**Disapprove/Not Support Use** No one disapproves Young people	**Approve/Support Use** Older people Most people/ everyone Family/ friends Irrelevant whether someone approves People who are active outside (e.g., doing work/ workouts)	**Disapprove/Not Support Use** No one disapproves Young people Irrelevant whether someone disapproves
**Likely to Use** Older people People with personal/family history of skin cancer Children People with fair skin/ prone to sunburn People who are active outside (e.g., doing work/ workouts) People who care about health/sun safety	**Unlikely to Use** Young people People who want to tan/ in the sun People who are active outside (e.g., doing work/ workouts) Those who don’t think/worry about sun risks	**Likely to Use** Older people People who are active outside (e.g., doing work/ workouts) Women	**Unlikely to Use** Young people People who are active outside (e.g., doing work/ workouts) Men People who don’t like hats/find hats uncomfortable/style-conscious people
***Control***			
**Facilitators/Easier to Use** Lightweight/cool/ comfortable Cool/breezy day/not humid day Reasonably priced/financial incentive Accessible/own/ remember to bring	**Barriers/Harder to Use** Hot/muggy When active Cost Not accessible/ don’t own	**Facilitators/Easier to Use** Properly fitting/ lighter/breathable comfortable Stylish/more options Stay on head Own/bought wide brimmed hat(s)	**Barriers/Harder to Use** Depends on wind/weather Doing physical activity/working outside Forgot to bring or put on Don’t own one Too hot/sweaty

### Using sunscreen

Nearly all participants reported that a positive of using sunscreen was reducing risk of sunburn and skin cancer and this was consistently reported across age and gender groups (Tables 4 and S1). Another commonly reported positive aspect was preventing aging and skin damage. Among a long list of aspects reported as bad/negative, those reported by the most individuals included sunscreen being greasy/sticky/messy, the application being time-consuming, and the need to re-apply; of note, some participants stated that there were no negative aspects of using sunscreen. Among judgments of who would approve of sunscreen use, ‘most people/everyone’, family/friends, healthcare providers, and parents/people with children were mostly commonly reported. With regard to perceived disapproval, the most commonly reported beliefs were that no one would disapprove of sunscreen use or that some young people would disapprove. People with fair skin/those who are prone to sunburn were most commonly believed to use sunscreen, in addition to parents/people with children, and children. ‘Young people’, men, and people with dark skin were commonly reported as unlikely to use sunscreen. The use of ‘young people’ versus ‘children’ among participants warrants a comment. Participants did not specify whom exactly they meant and how they differentiated between these groups. The way ‘young people’ was used left us with the impression that participants were referring to people old enough to take responsibility for their own sun behaviors, which may have conceptually included older teenagers under the age of 18, whereas the use of ‘children’, often used in conjunction with ‘parents’, suggested that sun protection decisions were being made for them. The commonly reported facilitators for sunscreen use were easily accessible, the type of sunscreen available (e.g., sprays, lotions), and ease of application. The most commonly reported barriers to sunscreen use were lack of access and forgetting to use.

### Staying in the shade

The most commonly provided positive aspects of staying in the shade included reduced risk of sunburn and/or skin cancer and protection from heat (Tables 4 and S2). A common response for negative aspects was ‘none’, though many participants reported negative aspects including not getting a suntan, being away from others and social activities, less vitamin D, bugs/insects, being cool, and not being able to work in the shade. Participants commonly reported friends/family, ‘most people/everyone’, and healthcare providers would support seeking shade to reduce sun exposure. In contrast, ‘no one’, young people, ‘people who want to be tan/in the sun’, and employers were most commonly believed to disapprove of seeking shade. Older people and people with fair skin or with a personal/family history of skin cancer were most commonly reported as unlikely to seek shade; whereas young people, ‘people who want to be tan/in the sun’ and people who are active outside (e.g., doing work/workouts) reported as being unlikely to seek shade. Availability of shade was the most commonly reported facilitator; lack of available shade, work requirements, and participation in activities were commonly reported barriers.

### Wearing protective clothing

Commonly reported beliefs about the positive aspects of wearing clothing to reduce sun exposure were reduced risk of sunburn and/or skin cancer and not having to use sunscreen (Tables 4 and S3). Sweating and overheating, feeling uncomfortable or skin irritation, and lack of a sun tan were commonly reported negative aspects. Groups frequently reported to be supportive of wearing protective clothing included family/friends, healthcare providers, older people, or ‘most people/everyone’, whereas ‘no one’ and young people were frequently reported as unsupportive. Older people, those with a personal or family history of skin cancer, children, and people with fair skin/prone to sunburn were commonly reported as being likely to use protective clothing; and young people, people who want to tan/enjoy the sun, and people who are physically active were frequently reported as not likely to use protective clothing. Common facilitators included lightweight/cool/comfortable clothing and it being a cool day; and common barriers included hot and/or muggy weather and being physically active.

### Wearing a wide-brimmed hat

Many participants mentioned reduced risk of sunburn and/or skin cancer, having the sun out of one’s eyes, and staying cool as positive aspects of wearing a wide-brimmed hat (Tables 4 and S4). Common reported negative aspects included that hats easily blow off and/or get lost, cause the head to get too warm and/or sweaty, are inconvenient and interfere with work, and not fashionable. Older people and ‘most people/everyone’ were commonly assumed to support use of hats; ‘no one’ or young people were often believed to disapprove. Older people, and people who are active outside were most commonly believed to wear a wide-brimmed hat; conversely people who are active outside were also among the most commonly reported groups to be unlikely to wear a wide-brimmed hat, in addition to young people. Commonly reported facilitators included if the hat fit properly, was light, breathable and comfortable; commonly reported barriers included windy or hot weather and being physically active outside.

## Discussion

Individuals living in rural Minnesota who participated in our study were aware of the protective effects of sun protective behaviors, with almost all participants reporting that each behavior would reduce risk of sunburn and skin cancer. Simultaneously, we found that for almost all participants, spending time outside in warm weather was important. Of the four sun protection behaviors, wearing protective clothing and using sunscreen were most consistently reported by study participants, although participants reported longer lists of negative than positive aspects of sunscreen use. Across all behaviors, many different beliefs were mentioned, but only a few salient beliefs were shared by almost everyone, with other beliefs only mentioned by a few participants. In addition to widespread awareness of reduced cancer and sunburn risks, salient beliefs across all behaviors included beliefs that most people would approve of anyone engaging in each protection behavior and that age was a factor in whether people were likely to adopt protective behaviors, with young people typically thought to be most unlikely to engage in each protective behavior. Further, many mentioned that being physically active such as doing outdoor work, was a factor for engagement in each behavior, albeit with varying directionality (for example, active people were assumed to stay in shade less often but wear a wide brimmed hat more often).

Almost all participants were aware of cancer and sunburn risks of sun exposures and reported barriers to engaging sun protective behaviors consistent with what has been reported for the general population, including not getting a tan, forgetting or being too busy to engage in the behavior, or not having the opportunity to engage in the behavior (e.g., because one did not own protective clothing, a wide brimmed hat, or because shade was not available) [45,46]. While future work will need to confirm similarities across rural and urban settings, our findings, in the context of prior work, suggest no substantial differences between our rural and the general population with regard to risk awareness and salient behavioral barriers. Of note, negative judgement from others about each behavior was not a frequently reported barrier in our rural study, with many participants saying that few people, if anyone, would not support a given behavior or that support by others or lack thereof was irrelevant.

The finding that our participants were aware of sun exposures and did not typically have concerns about others judging them for engaging in a given behavior further suggests that the reasons that some individuals living in rural areas do not engage in some sun protection behaviors are not primarily belief-based. Rural barriers to sun protection behaviors may be practical instead; many of our participants stated that they simply could not engage in a given behavior if they wanted to be able to do what mattered to them (e.g., because there was work or an activity to be done outside that would conflict with protective behaviors), either because the protection method was inconvenient (e.g., shade, hat), or because they did not have the means to engage in that behavior (e.g., not owning sun protective hat, lack of available shade). If confirmed in future research, this finding would suggest that rural sun behavior intervention strategies may need to focus on addressing rural circumstances that either facilitate sun protection behaviors or make them harder. For example, one question arising from this finding is how effective sun protection measures can smoothly be incorporated into the realities of rural residents who work outdoors, potentially necessitating structural changes that would make safe sun behaviors more easily accessible, for example by making sun protective clothing and hats more affordable and available in rural areas, or through work regulations to promote sun safety behaviors among outdoor workers.

Our finding that sunscreen was the most consistently used protection behavior somewhat contrasts with national data where sunscreen was the second common protection behavior, following staying in the shade [24]. Reasons for this difference could be Minnesota specific-circumstances, i.e., hotter temperatures in other parts of the country may promote more use of shade. An additional finding that may be region- and less rural-specific was that most participants reported that spending time outside was important to them. Spending time outdoors may be more relevant in states with colder climates where summers are shorter, with a possible greater urge to spend time outside when it is warm. In rural areas, the importance of spending time outdoors may be especially important, further aggravating potential regional patterns that may add to sun risks. Together, these findings suggest that rural characteristics that drive sun protection behaviors may be influenced by regional characteristics, meaning that potential sun protection interventions would need to account for regional and rural contexts simultaneously.

Despite sunscreen being among the most frequently reported sun protective behavior, participants cited many negative aspects of sunscreen use, which seems somewhat paradoxical. It is possible that because sunscreen is a common behavior, people are also most acutely aware of its downsides. Another possible explanation is that although shorter lists of negative aspects were reported for other behaviors, these negative aspects were more prohibitive against engaging in the behavior. For example, salient concerns about the least frequently reported behaviors, i.e., staying in the shade and wearing a wide-brimmed hat, included hats blowing away or being too hot or a nuisance, and no availability of shade near their planned outdoor activity.

Limitations of our study included lack of racial diversity, with most of our participants being non-Hispanic White, however this demographic composition is largely reflective of rural Minnesota. Further, long cold winters may translate into different sun protective and risk behaviors than in states with longer and warmer summers, limiting the generalizability of our findings. An additional limitation is the low response rate. This initial qualitative study was meant to inform survey development for further in-depth research to obtain frequencies of these beliefs. We approached rural residents across Minnesota identified from purchased address lists. A more targeted approach (e.g., by collaborating with dermatologists) may have resulted in higher response rates, but may have biased the sample toward those who are most concerned about skin health. While our approach to throw a wide net for recruitment resulted in low response rates, participants may be more likely to represent average beliefs about skin health. Further, we did not include an urban comparison group, therefore we cannot determine which of our findings are specific to rural populations and which are pertinent to urban populations as well. However, given well-documented rural health disparities, our results provide much-needed information on how to target approaches to rural residents. Finally, the anchor for our questions was “on a typical sunny day in the summer”, which does not allow us to understand beliefs about sun protective behaviors on cloudy days in the summer or sunny days outside of summer.

## Conclusion

Public health interventions to reduce sun exposures may need to account for rural as well as regional circumstances and address how effective sun protection methods can be made easily and structurally accessible to rural populations without impeding lifestyle.

## Supporting information

S1 Fig Average number of positive and negative outcome beliefs reported for using sunscreen by gender and age group. (TIF)

S2 Fig Average number of positive and negative outcome beliefs reported for staying in the shade by gender and age group. (TIF)

S3 Fig Average number of positive and negative outcome beliefs reported for wearing protective clothing by gender and age group. (TIF)

S4 Fig Average number of positive and negative outcome beliefs reported for wearing a wide-brimmed hat by gender and age group. (TIF)

S1 Table All reported beliefs about using sunscreen to reduce sun exposure and prevent sunburn on typical sunny day in the summer. (DOCX)

S2 Table All reported beliefs about seeking shade to reduce sun exposure and prevent sunburn on typical sunny day in the summer. (DOCX)

S3 Table All reported beliefs about wearing clothing to reduce sun exposure and prevent sunburn on typical sunny day in the summer. (DOCX)

S4 Table All beliefs about wearing a hat to reduce sun exposure and prevent sunburn on typical sunny day in the summer. (DOCX)

## References

[1] Linos E, Katz KA, Colditz GA. Skin cancer-the importance of prevention. JAMA Intern Med. 2016;176(10):1435–6. doi: 10.1001/jamainternmed.2016.5008 27459394 PMC5489348

[2] Brunssen A, Waldmann A, Eisemann N, Katalinic A. Impact of skin cancer screening and secondary prevention campaigns on skin cancer incidence and mortality: a systematic review. J Am Acad Dermatol. 2017;76(1):129-139.e10. doi: 10.1016/j.jaad.2016.07.045 27707591

[3] American Cancer Society. Cancer facts and figures 2025. Atlanta (GA): American Cancer Society; 2025.

[4] Gilchrest BA, Eller MS, Geller AC, Yaar M. The pathogenesis of melanoma induced by ultraviolet radiation. N Engl J Med. 1999;340(17):1341–8. doi: 10.1056/NEJM199904293401707 10219070

[5] Narayanan DL, Saladi RN, Fox JL. Ultraviolet radiation and skin cancer. Int J Dermatol. 2010;49(9):978–86. doi: 10.1111/j.1365-4632.2010.04474.x 20883261

[6] Gandini S, Sera F, Cattaruzza MS, Pasquini P, Picconi O, Boyle P, et al. Meta-analysis of risk factors for cutaneous melanoma: II. Sun exposure. Eur J Cancer. 2005;41(1):45–60. doi: 10.1016/j.ejca.2004.10.016 15617990

[7] Holman DM, Ding H, Berkowitz Z, Hartman AM, Perna FM. Sunburn prevalence among US adults, National Health Interview Survey 2005, 2010, and 2015. J Am Acad Dermatol. 2019;80(3):817–20. doi: 10.1016/j.jaad.2018.10.044 30744879 PMC6681661

[8] Zahnd WE, James AS, Jenkins WD, Izadi SR, Fogleman AJ, Steward DE, et al. Rural–urban differences in cancer incidence and trends in the United States. AACR; 2018. 10.1158/1055-9965.EPI-17-0430 PMC5787045 28751476

[9] Mounessa JS, Caravaglio JV, Dellavalle RP. Comparison of regional and state differences in melanoma rates in the United States: 2003 vs 2013. JAMA Dermatol. 2017;153(3):345–7. doi: 10.1001/jamadermatol.2016.4625 28030665

[10] Islami F, Sauer AG, Miller KD, Fedewa SA, Minihan AK, Geller AC, et al. Cutaneous melanomas attributable to ultraviolet radiation exposure by state. Int J Cancer. 2020;147(5):1385–90. doi: 10.1002/ijc.32921 32064604

[11] Holman DM, Ding H, Berkowitz Z, Hartman AM, Perna FM. Sunburn prevalence among US adults, National Health Interview Survey 2005, 2010, and 2015. J Am Acad Dermatol. 2019;80(3):817–20. 30744879 10.1016/j.jaad.2018.10.044 PMC6681661

[12] Blake KD, Moss JL, Gaysynsky A, Srinivasan S, Croyle RT. Making the case for investment in rural cancer control: an analysis of rural cancer incidence, mortality, and funding trends. Cancer Epidemiol Biomarkers Prev. 2017;26(7):992–7. doi: 10.1158/1055-9965.EPI-17-0092 28600296 PMC5500425

[13] Greater minnesota refined & revisited. St. Paul (MN): Minnesota State Demographic Center; 2017. Report No.

[14] Centers for Disease Control and Prevention. Sun safety facts. Centers for Disease Control and Prevention; 2024 [cited 2025 Feb 13]. Available from: https://www.cdc.gov/skin-cancer/sun-safety/index.html

[15] Zahnd WE, Goldfarb J, Scaife SL, Francis ML. Rural-urban differences in behaviors to prevent skin cancer: an analysis of the Health Information National Trends Survey. J Am Acad Dermatol. 2010;62(6):950–6. doi: 10.1016/j.jaad.2009.08.058 20236728

[16] Nagelhout ES, Parsons BG, Haaland B, Tercyak KP, Zaugg K, Zhu A, et al. Differences in reported sun protection practices, skin cancer knowledge, and perceived risk for skin cancer between rural and urban high school students. Cancer Causes Control. 2019;30(11):1251–8. doi: 10.1007/s10552-019-01228-5 31522321 PMC6802938

[17] Cunningham SA, Yu R, Shete S. Differences in sun protection behaviors between rural and urban communities in Texas. J Rural Health. 2019;35(2):155–66. doi: 10.1111/jrh.12350 30830988 PMC6436991

[18] Vogel RI, Jewett PI, Ahmed RL, Lazovich D. Comparison of sun exposure and protection behaviors between urban and rural residents without a history of melanoma in the Midwestern United States. J Am Acad Dermatol. 2022;86(1):229–32. doi: 10.1016/j.jaad.2021.01.095 33716058 PMC8433256

[19] Dona AC, Jewett PI, Henning-Smith C, Ahmed RL, Wei ML, Lazovich D, et al. Rural-urban differences in sun exposure and protection behaviors in the United States. Cancer Epidemiol Biomarkers Prev. 2024;33(4):608–15. doi: 10.1158/1055-9965.EPI-23-1264 38227023 PMC10990774

[20] Zafar FS, Abid R, Ginader T, Powers JG. Rural health disparities in melanoma staging and prognostic outcomes in Iowa. J Am Acad Dermatol. 2021;84(6):1727–30. doi: 10.1016/j.jaad.2020.08.092 32860920

[21] Odahowski CL, Zahnd WE, Zgodic A, Edward JS, Hill LN, Davis MM, et al. Financial hardship among rural cancer survivors: an analysis of the Medical Expenditure Panel Survey. Prev Med. 2019;129S:105881. doi: 10.1016/j.ypmed.2019.105881 31727380 PMC7190004

[22] Volkov A, Dobbinson S, Wakefield M, Slevin T. Seven-year trends in sun protection and sunburn among Australian adolescents and adults. Aust N Z J Public Health. 2013;37(1):63–9. doi: 10.1111/1753-6405.12012 23379808

[23] Holman DM, Berkowitz Z, Guy GP Jr, Hartman AM, Perna FM. The association between demographic and behavioral characteristics and sunburn among U.S. adults - National Health Interview Survey, 2010. Prev Med. 2014;63:6–12. doi: 10.1016/j.ypmed.2014.02.018 24589442 PMC4535173

[24] Holman DM, Ding H, Guy GP Jr, Watson M, Hartman AM, Perna FM. Prevalence of sun protection use and sunburn and association of demographic and behaviorial characteristics with sunburn among US adults. JAMA Dermatol. 2018;154(5):561–8. doi: 10.1001/jamadermatol.2018.0028 29541756 PMC5876912

[25] Holman DM, Ragan KR, Julian AK, Perna FM. The context of sunburn among U.S. adults: common activities and sun protection behaviors. Am J Prev Med. 2021;60(5):e213–20. doi: 10.1016/j.amepre.2020.12.011 33589300 PMC8068601

[26] Wu YP, Parsons B, Jo Y, Chipman J, Haaland B, Nagelhout ES, et al. Outdoor activities and sunburn among urban and rural families in a Western region of the US: implications for skin cancer prevention. Prev Med Rep. 2022;29:101914. doi: 10.1016/j.pmedr.2022.101914 35911574 PMC9326324

[27] Fishbein M, Ajzen I. Predicting and changing behavior: the reasoned action approach. New York: Psychology Press; 2010.

[28] Starfelt Sutton LC, White KM. Predicting sun-protective intentions and behaviours using the theory of planned behaviour: a systematic review and meta-analysis. Psychol Health. 2016;31(11):1272–92. doi: 10.1080/08870446.2016.1204449 27334551

[29] Fishbein M, Yzer MC. Using theory to design effective health behavior interventions. Commun Theory. 2003;13(2):164–83. doi: 10.1111/j.1468-2885.2003.tb00287.x

[30] Fishbein M, Cappella JN. The role of theory in developing effective health communications. J Commun. 2006;56(suppl_1):S1–17. doi: 10.1111/j.1460-2466.2006.00280.x

[31] Epton T, Norman P, Harris P, Webb T, Snowsill FA, Sheeran P. Development of theory-based health messages: three-phase programme of formative research. Health Promot Int. 2015;30(3):756–68. doi: 10.1093/heapro/dau005 24504361 PMC4542920

[32] Hillhouse JJ, Stair AW 3rd, Adler CM. Predictors of sunbathing and sunscreen use in college undergraduates. J Behav Med. 1996;19(6):543–61. doi: 10.1007/BF01904903 8970914

[33] Thieden E, Philipsen PA, Sandby-Møller J, Wulf HC. Sunscreen use related to UV exposure, age, sex, and occupation based on personal dosimeter readings and sun-exposure behavior diaries. Arch Dermatol. 2005;141(8):967–73. doi: 10.1001/archderm.141.8.967 16103325

[34] Wichstrøm L. Predictors of Norwegian adolescents’ sunbathing and use of sunscreen. Health Psychol. 1994;13(5):412–20. 7805636

[35] Arthey S, Clarke VA. Suntanning and sun protection: a review of the psychological literature. Soc Sci Med. 1995;40(2):265–74. doi: 10.1016/0277-9536(94)e0063-x 7899938

[36] Bleakley A, Jordan AB, Strasser AA, Lazovich D, Glanz K. Testing general versus specific behavioral focus in messaging for the promotion of sun protection behaviors. Ann Behav Med. 2020;54(2):108–18. doi: 10.1093/abm/kaz032 31586204

[37] Middlestadt SE. Beliefs underlying eating better and moving more. Ann Am Acad Pol Soc Sci. 2012;640(1):81–100. doi: 10.1177/0002716211425015

[38] Erbe RG, Middlestadt SE, Lohrmann DK, Beckmeyer JJ. A salient belief elicitation examining adolescents’ meditation beliefs using the reasoned action approach. Health Promot Pract. 2020;21(4):633–41. doi: 10.1177/1524839918811803 30442018

[39] Owens C, Le AB, Smith TD, Middlestadt SE. Beliefs of university employees leaving during a fire alarm: a theory-based belief elicitation. Saf Health Work. 2023;14(2):201–6. doi: 10.1016/j.shaw.2023.03.002 37389314 PMC10300468

[40] Dillman D, Smith J, Christian L. Internet, phone, mail and mixed-mode surveys: the tailored design method. 4th ed. Hoboken (NJ): Wiley and Sons, Inc.; 2014.

[41] Harris PA, Taylor R, Thielke R, Payne J, Gonzalez N, Conde JG. Research electronic data capture (REDCap)--a metadata-driven methodology and workflow process for providing translational research informatics support. J Biomed Inform. 2009;42(2):377–81. doi: 10.1016/j.jbi.2008.08.010 18929686 PMC2700030

[42] Middlestadt SE, Bhattacharyya K, Rosenbaum J, Fishbein M, Shepherd M. The use of theory based semistructured elicitation questionnaires: formative research for CDC’s Prevention Marketing Initiative. Public Health Rep. 1996;111 Suppl 1(Suppl 1):18–27. 8862153 PMC1382039

[43] Glanz K, Yaroch AL, Dancel M, Saraiya M, Crane LA, Buller DB, et al. Measures of sun exposure and sun protection practices for behavioral and epidemiologic research. Arch Dermatol. 2008;144(2):217–22. doi: 10.1001/archdermatol.2007.46 18283179

[44] Yzer M, Zhu X. A research protocol for determining depression help-seeking message content. Yzer M, Siegel JT, editors. Wiley; In press.

[45] Kirk L, Greenfield S. Knowledge and attitudes of UK university students in relation to ultraviolet radiation (UVR) exposure and their sun-related behaviours: a qualitative study. BMJ Open. 2017;7(3):e014388. doi: 10.1136/bmjopen-2016-014388 28289050 PMC5353347

[46] Dennis LK, Lowe JB, Snetselaar LG. Tanning behavior among young frequent tanners is related to attitudes and not lack of knowledge about the dangers. Health Educ J. 2009;68(3):232–43. doi: 10.1177/0017896909345195 22707763 PMC3374486

