# Supplementary figures and images for: Sun protection behavior beliefs among adults living in rural United States: A qualitative study in Minnesota
Source: PLoS One. 2025 Sep 12;20(9):e0331685. doi: 10.1371/journal.pone.0331685 (PMC12431656; doi:10.1371/journal.pone.0331685)

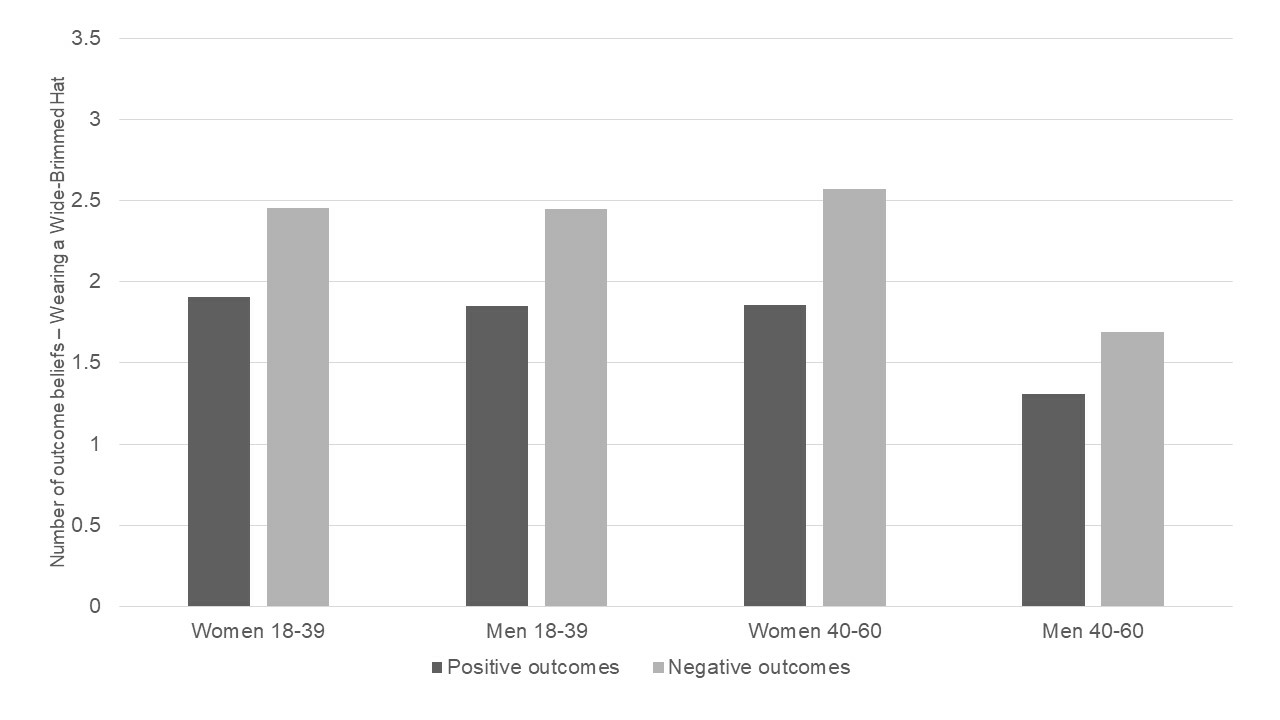

Supplement: S1 Fig — (TIF) [file pone.0331685.s001.tif]

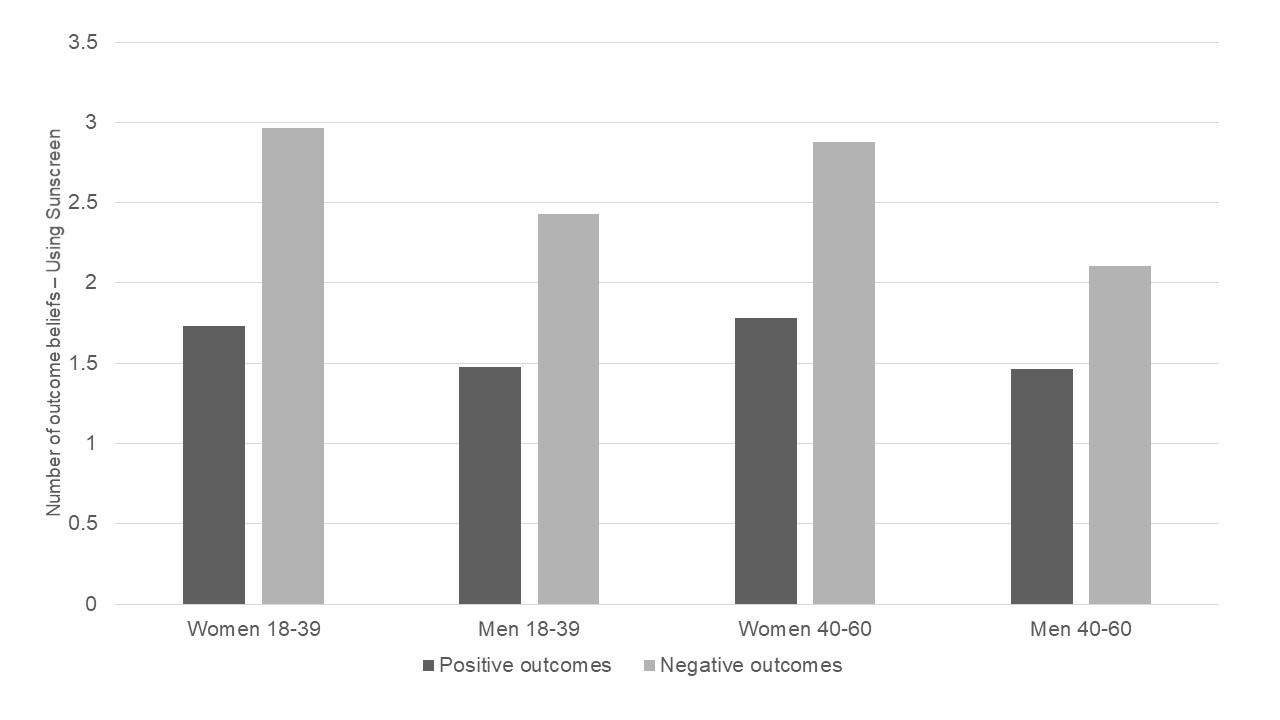

Supplement: S2 Fig — (TIF) [file pone.0331685.s002.tif]

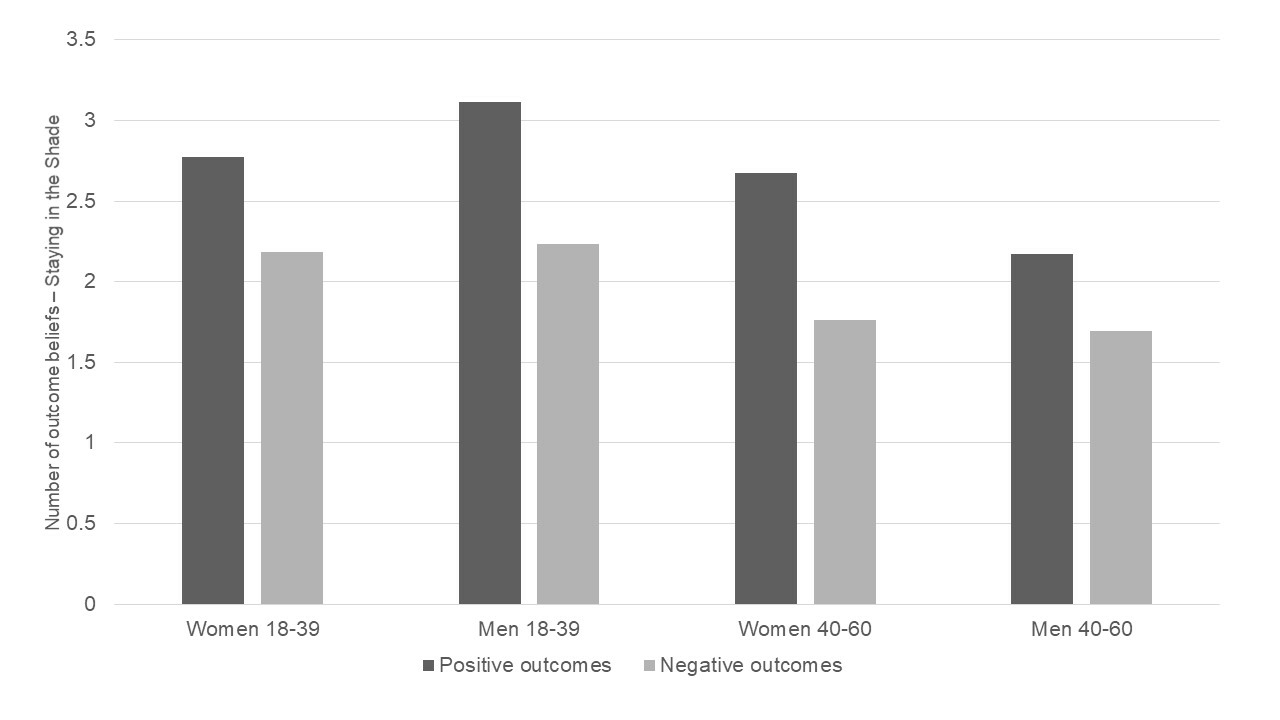

Supplement: S3 Fig — (TIF) [file pone.0331685.s003.tif]

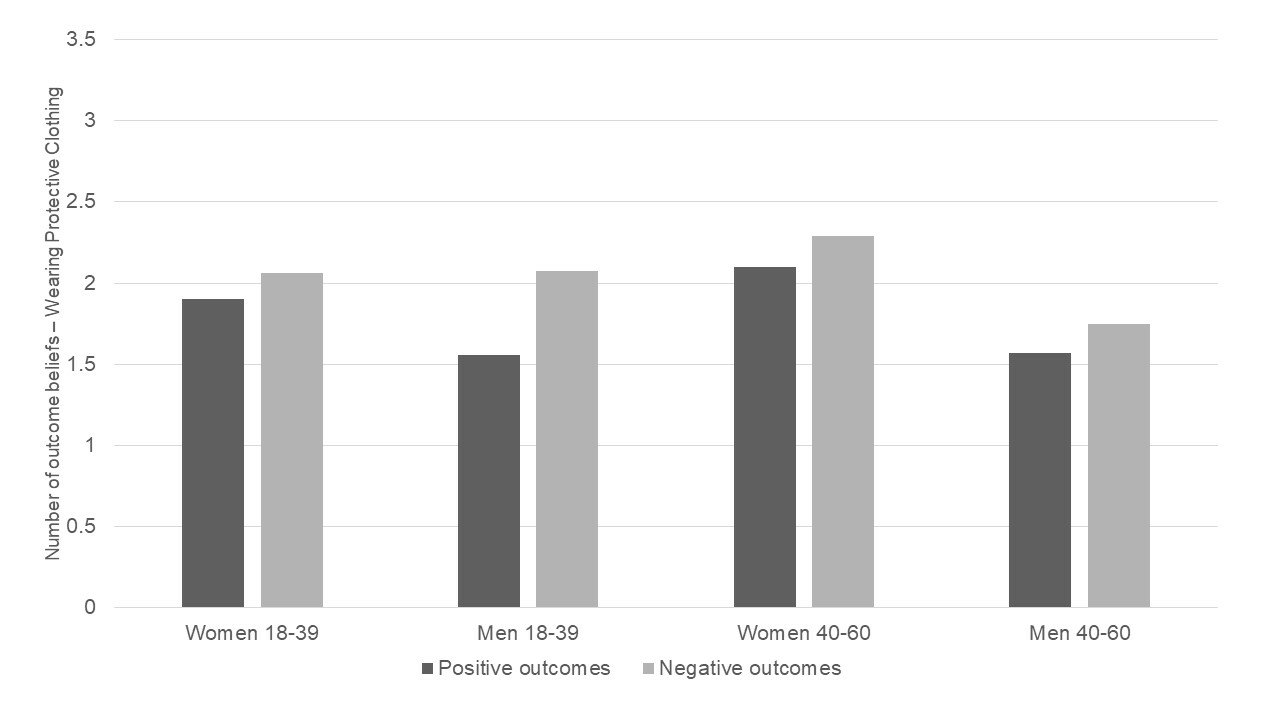

Supplement: S4 Fig — (TIF) [file pone.0331685.s004.tif]
