# Supplementary material for: Sun protection behavior beliefs among adults living in rural United States: A qualitative study in Minnesota
Source: PLoS One. 2025 Sep 12;20(9):e0331685. doi: 10.1371/journal.pone.0331685 (PMC12431656; doi:10.1371/journal.pone.0331685)
Supplement: S1 Table — (DOCX) [file pone.0331685.s005.docx]

**S1 Table. All reported beliefs about using sunscreen to reduce sun exposure and prevent sunburn on typical sunny day in the summer.**

|  | **Overall**  **N=111** | | **18-39 Female**  **N=30** | | **18-39 Male**  **N=21** | | **40-60 Female**  **N=32** | | **40-60 Male**  **N=28** | |
| --- | --- | --- | --- | --- | --- | --- | --- | --- | --- | --- |
| **Beliefs** | **n** | **%** | **n** | **%** | **n** | **%** | **n** | **%** | **n** | **%** |
| ***Outcome*** |  |  |  |  |  |  |  |  |  |  |
| **Good/Positive** |  |  |  |  |  |  |  |  |  |  |
| Less cancer / exposure / sunburn | 109 | 98.2 | 30 | 100.0 | 20 | 95.2 | 31 | 96.9 | 28 | 100.0 |
| Prevent aging / less skin damage | 24 | 21.6 | 9 | 30.0 | 2 | 9.5 | 9 | 28.1 | 4 | 14.3 |
| Keep skin healthy / healthy choice / be proactive | 16 | 14.4 | 6 | 20.0 | 2 | 9.5 | 5 | 15.6 | 3 | 10.7 |
| Stay outside longer | 6 | 5.4 | 2 | 6.7 | 0 | 0.0 | 3 | 9.4 | 1 | 3.6 |
| Set example for kids / family | 5 | 4.5 | 1 | 3.3 | 0 | 0.0 | 4 | 12.5 | 0 | 0.0 |
| Confident skin is protected / reduce anxiety about sun exposure | 4 | 3.6 | 1 | 3.3 | 1 | 4.8 | 2 | 6.3 | 0 | 0.0 |
| Moisturize skin | 3 | 2.7 | 0 | 0.0 | 0 | 0.0 | 2 | 6.3 | 1 | 3.6 |
| Even skin color / reduce tan lines | 3 | 2.7 | 2 | 6.7 | 0 | 0.0 | 0 | 0.0 | 1 | 3.6 |
| Nothing positive about sunscreen | 3 | 2.7 | 1 | 3.3 | 2 | 9.5 | 0 | 0.0 | 0 | 0.0 |
| Stay cooler | 2 | 1.8 | 0 | 0.0 | 1 | 4.8 | 0 | 0.0 | 1 | 3.6 |
| Smells good | 2 | 1.8 | 0 | 0.0 | 0 | 0.0 | 1 | 3.1 | 1 | 3.6 |
| Don't have to stay in the shade / wear protective clothing | 2 | 1.8 | 0 | 0.0 | 2 | 9.5 | 0 | 0.0 | 0 | 0.0 |
| Less dehydrated | 1 | 0.9 | 0 | 0.0 | 1 | 4.8 | 0 | 0.0 | 0 | 0.0 |
| Protect hair | 1 | 0.9 | 0 | 0.0 | 0 | 0.0 | 0 | 0.0 | 1 | 3.6 |
| **Bad/Negative** |  |  |  |  |  |  |  |  |  |  |
| Greasy / sticky / messy | 56 | 50.5 | 19 | 63.3 | 9 | 42.9 | 15 | 46.9 | 13 | 46.4 |
| Nothing negative about sunscreen | 32 | 28.8 | 7 | 23.3 | 6 | 28.6 | 8 | 25.0 | 11 | 39.3 |
| Time consuming / Not easy to get all areas covered / miss areas / Difficult to apply | 28 | 25.2 | 6 | 20.0 | 10 | 47.6 | 9 | 28.1 | 3 | 10.7 |
| Need to reapply | 21 | 18.9 | 6 | 20.0 | 4 | 19.1 | 8 | 25.0 | 3 | 10.7 |
| Chemicals / Don't know ingredients | 16 | 14.4 | 7 | 23.3 | 3 | 14.3 | 3 | 9.4 | 3 | 10.7 |
| Inconvenient / annoying / Need to remember to bring / carry it | 16 | 14.4 | 3 | 10.0 | 4 | 19.1 | 7 | 21.9 | 2 | 7.1 |
| No suntan / pale skin | 16 | 14.4 | 6 | 20.0 | 3 | 14.3 | 6 | 18.8 | 1 | 3.6 |
| Gets in eyes / burns eyes | 13 | 11.7 | 2 | 6.7 | 3 | 14.3 | 5 | 15.6 | 3 | 10.7 |
| Irritates skin / acne / allergic reaction | 13 | 11.7 | 7 | 23.3 | 0 | 0.0 | 4 | 12.5 | 2 | 7.1 |
| Expensive / cost | 10 | 9.0 | 3 | 10.0 | 3 | 14.3 | 3 | 9.4 | 1 | 3.6 |
| Stains clothes / things you touch / leaves residue | 10 | 9.0 | 3 | 10.0 | 1 | 4.8 | 3 | 9.4 | 3 | 10.7 |
| Sweating it off | 10 | 9.0 | 2 | 6.7 | 2 | 9.5 | 3 | 9.4 | 3 | 10.7 |
| Sand/dirt/bugs stick to it | 7 | 6.3 | 0 | 0.0 | 1 | 4.8 | 4 | 12.5 | 2 | 7.1 |
| Bad smell | 7 | 6.3 | 2 | 6.7 | 0 | 0.0 | 3 | 9.4 | 2 | 7.1 |
| Harm animals / pollution | 6 | 5.4 | 3 | 10.0 | 1 | 4.8 | 0 | 0.0 | 2 | 7.1 |
| Hard to find good brands at local store / Multiple types / confusing / Not sure using right sunscreen / which SPF to use / how effective | 5 | 4.5 | 2 | 6.7 | 1 | 4.8 | 2 | 6.3 | 0 | 0.0 |
| Need to apply it in advance | 4 | 3.6 | 1 | 3.3 | 0 | 0.0 | 3 | 9.4 | 0 | 0.0 |
| Hot skin / Keeps skin from breathing | 4 | 3.6 | 1 | 3.3 | 0 | 0.0 | 1 | 3.1 | 2 | 7.1 |
| Doesn't work well in water | 3 | 2.7 | 3 | 10.0 | 0 | 0.0 | 0 | 0.0 | 0 | 0.0 |
| Sunscreen expires | 3 | 2.7 | 1 | 3.3 | 0 | 0.0 | 2 | 6.3 | 0 | 0.0 |
| Scent attracts bugs | 2 | 1.8 | 1 | 3.3 | 0 | 0.0 | 1 | 3.1 | 0 | 0.0 |
| Doesn't blend in skin | 2 | 1.8 | 1 | 3.3 | 0 | 0.0 | 0 | 0.0 | 1 | 3.6 |
| Not enough sun exposure | 2 | 1.8 | 1 | 3.3 | 0 | 0.0 | 0 | 0.0 | 1 | 3.6 |
| Need to wash off / Need to shower afterwards | 2 | 1.8 | 1 | 3.3 | 0 | 0.0 | 1 | 3.1 | 0 | 0.0 |
| Don't like it | 1 | 0.9 | 0 | 0.0 | 0 | 0.0 | 1 | 3.1 | 0 | 0.0 |
| Glasses slip off | 1 | 0.9 | 1 | 3.3 | 0 | 0.0 | 0 | 0.0 | 0 | 0.0 |
| Unavailable | 1 | 0.9 | 0 | 0.0 | 0 | 0.0 | 0 | 0.0 | 1 | 3.6 |
| ***Normative*** |  |  |  |  |  |  |  |  |  |  |
| **Approve/Support Use** |  |  |  |  |  |  |  |  |  |  |
| Most people / everyone | 39 | 35.1 | 11 | 36.7 | 10 | 47.6 | 10 | 31.3 | 8 | 28.6 |
| Family / Friends | 37 | 33.3 | 7 | 23.4 | 7 | 33.4 | 12 | 37.5 | 11 | 39.3 |
| Healthcare providers | 21 | 18.9 | 5 | 16.7 | 4 | 19.1 | 8 | 25.0 | 4 | 14.3 |
| Parents/people with children | 14 | 12.6 | 4 | 13.3 | 1 | 4.8 | 7 | 21.9 | 2 | 7.1 |
| People aware of effects of sun exposure | 8 | 7.2 | 1 | 3.3 | 2 | 9.5 | 3 | 9.4 | 2 | 7.1 |
| Older people | 6 | 5.4 | 4 | 13.3 | 1 | 4.8 | 1 | 3.1 | 0 | 0.0 |
| People with personal or family history of skin cancer | 6 | 5.4 | 2 | 6.7 | 1 | 4.8 | 3 | 9.4 | 0 | 0.0 |
| People with pale/fair skin | 4 | 3.6 | 0 | 0.0 | 1 | 4.8 | 1 | 3.1 | 2 | 7.1 |
| People afraid of sun / skin cancer / paranoid | 3 | 2.7 | 1 | 3.3 | 0 | 0.0 | 1 | 3.1 | 1 | 3.6 |
| Don't care / let people do what they want / irrelevant question | 3 | 2.7 | 1 | 3.3 | 0 | 0.0 | 1 | 3.1 | 1 | 3.6 |
| Beach goers / lifeguards | 2 | 1.8 | 1 | 3.3 | 0 | 0.0 | 0 | 0.0 | 1 | 3.6 |
| Smart people / better educated | 2 | 1.8 | 0 | 0.0 | 0 | 0.0 | 1 | 3.1 | 1 | 3.6 |
| Women | 2 | 1.8 | 0 | 0.0 | 1 | 4.8 | 1 | 3.1 | 0 | 0.0 |
| Healthy people | 2 | 1.8 | 1 | 3.3 | 0 | 0.0 | 1 | 3.1 | 0 | 0.0 |
| No one cares | 2 | 1.8 | 2 | 6.7 | 0 | 0.0 | 0 | 0.0 | 0 | 0.0 |
| People who are patient | 1 | 0.9 | 0 | 0.0 | 0 | 0.0 | 1 | 3.1 | 0 | 0.0 |
| People who work outside | 1 | 0.9 | 0 | 0.0 | 1 | 4.8 | 0 | 0.0 | 0 | 0.0 |
| Don't know | 1 | 0.9 | 0 | 0.0 | 0 | 0.0 | 1 | 3.1 | 0 | 0.0 |
| Scientists | 1 | 0.9 | 0 | 0.0 | 0 | 0.0 | 1 | 3.1 | 0 | 0.0 |
| Hippies / 'woke' people | 1 | 0.9 | 0 | 0.0 | 1 | 4.8 | 0 | 0.0 | 0 | 0.0 |
| City people | 1 | 0.9 | 0 | 0.0 | 0 | 0.0 | 0 | 0.0 | 1 | 3.6 |
| Insurance companies | 1 | 0.9 | 0 | 0.0 | 0 | 0.0 | 1 | 3.1 | 0 | 0.0 |
| Sunscreen companies | 1 | 0.9 | 0 | 0.0 | 0 | 0.0 | 0 | 0.0 | 1 | 3.6 |
| Young people | 1 | 0.9 | 1 | 3.3 | 0 | 0.0 | 0 | 0.0 | 0 | 0.0 |
| **Disapprove/Not Support Use** |  |  |  |  |  |  |  |  |  |  |
| No one disapproves | 57 | 51.4 | 14 | 46.7 | 12 | 57.1 | 16 | 50.0 | 15 | 53.6 |
| Young people | 10 | 9.0 | 4 | 13.3 | 0 | 0.0 | 5 | 15.6 | 1 | 3.6 |
| Conservationists / naturalists | 8 | 7.2 | 3 | 10.0 | 1 | 4.8 | 1 | 3.1 | 3 | 10.7 |
| People who want to be tan / in the sun | 6 | 5.4 | 1 | 3.3 | 2 | 9.5 | 1 | 3.1 | 2 | 7.1 |
| People not concerned about risk of sunburn | 5 | 4.5 | 0 | 0.0 | 3 | 14.3 | 2 | 6.3 | 0 | 0.0 |
| Uneducated people | 4 | 3.6 | 1 | 3.3 | 1 | 4.8 | 1 | 3.1 | 1 | 3.6 |
| Don't care | 4 | 3.6 | 0 | 0.0 | 0 | 0.0 | 2 | 6.3 | 2 | 7.1 |
| Rednecks / Antivaxers / People who want to be 'tough' | 3 | 2.7 | 0 | 0.0 | 1 | 4.8 | 0 | 0.0 | 2 | 7.1 |
| Don't know | 3 | 2.7 | 1 | 3.3 | 0 | 0.0 | 1 | 3.1 | 1 | 3.6 |
| People in a hurry / impatient to take time to apply | 2 | 1.8 | 1 | 3.3 | 0 | 0.0 | 1 | 3.1 | 0 | 0.0 |
| Don't want to use chemicals / concerned about ingredients | 2 | 1.8 | 1 | 3.3 | 0 | 0.0 | 1 | 3.1 | 0 | 0.0 |
| Family / specific family members | 2 | 1.8 | 0 | 0.0 | 1 | 4.8 | 1 | 3.1 | 0 | 0.0 |
| Young females | 2 | 1.8 | 0 | 0.0 | 1 | 4.8 | 0 | 0.0 | 1 | 3.6 |
| Older people | 2 | 1.8 | 2 | 6.7 | 0 | 0.0 | 0 | 0.0 | 0 | 0.0 |
| People who don't like sunscreen | 2 | 1.8 | 1 | 3.3 | 1 | 4.8 | 0 | 0.0 | 0 | 0.0 |
| Men | 1 | 0.9 | 0 | 0.0 | 0 | 0.0 | 1 | 3.1 | 0 | 0.0 |
| Tanning salon owners | 1 | 0.9 | 1 | 3.3 | 0 | 0.0 | 0 | 0.0 | 0 | 0.0 |
| People who don't know benefits of using sunscreen | 1 | 0.9 | 0 | 0.0 | 1 | 4.8 | 0 | 0.0 | 0 | 0.0 |
| Low income | 1 | 0.9 | 1 | 3.3 | 0 | 0.0 | 0 | 0.0 | 0 | 0.0 |
| Blue collar males | 1 | 0.9 | 0 | 0.0 | 0 | 0.0 | 0 | 0.0 | 1 | 3.6 |
| **Likely to Use** |  |  |  |  |  |  |  |  |  |  |
| People with fair skin / prone to sunburn | 32 | 28.8 | 7 | 23.3 | 9 | 42.9 | 9 | 28.1 | 7 | 25.0 |
| Parents/people with children | 28 | 25.2 | 11 | 36.7 | 4 | 19.1 | 10 | 31.3 | 3 | 10.7 |
| Children | 25 | 22.5 | 6 | 20.0 | 6 | 28.6 | 8 | 25.0 | 5 | 17.9 |
| Older people | 23 | 20.7 | 8 | 26.7 | 6 | 28.6 | 5 | 15.6 | 4 | 14.3 |
| People who have personal or family history of skin cancer | 12 | 10.8 | 3 | 10.0 | 2 | 9.5 | 5 | 15.6 | 2 | 7.1 |
| People swimming / at a beach / pool | 9 | 8.1 | 4 | 13.3 | 0 | 0.0 | 4 | 12.5 | 1 | 3.6 |
| People educated on skin cancer risks | 9 | 8.1 | 1 | 3.3 | 0 | 0.0 | 5 | 15.6 | 3 | 10.7 |
| People concerned with health | 9 | 8.1 | 1 | 3.3 | 2 | 9.5 | 3 | 9.4 | 3 | 10.7 |
| Women | 8 | 7.2 | 3 | 10.0 | 2 | 9.5 | 3 | 9.4 | 0 | 0.0 |
| Middle age adults | 7 | 6.3 | 4 | 13.3 | 2 | 9.5 | 0 | 0.0 | 1 | 3.6 |
| Outdoorsy people (hikers, fishermen, golfers, tourists) | 6 | 5.4 | 3 | 10.0 | 1 | 4.8 | 0 | 0.0 | 2 | 7.1 |
| People who care about looks / aging / sun spots | 5 | 4.5 | 2 | 6.7 | 0 | 0.0 | 2 | 6.3 | 1 | 3.6 |
| People who spend time outside | 4 | 3.6 | 1 | 3.3 | 0 | 0.0 | 0 | 0.0 | 3 | 10.7 |
| Most people / everyone | 4 | 3.6 | 1 | 3.3 | 1 | 4.8 | 1 | 3.1 | 1 | 3.6 |
| Athletes | 4 | 3.6 | 1 | 3.3 | 0 | 0.0 | 2 | 6.3 | 1 | 3.6 |
| Responsible people / people with common sense | 3 | 2.7 | 0 | 0.0 | 0 | 0.0 | 2 | 6.3 | 1 | 3.6 |
| Healthcare providers | 3 | 2.7 | 0 | 0.0 | 0 | 0.0 | 3 | 9.4 | 0 | 0.0 |
| People who work outside | 3 | 2.7 | 1 | 3.3 | 2 | 9.5 | 0 | 0.0 | 0 | 0.0 |
| Planning to spend a long time outside | 2 | 1.8 | 0 | 0.0 | 0 | 0.0 | 1 | 3.1 | 1 | 3.6 |
| People who work with children | 2 | 1.8 | 1 | 3.3 | 1 | 4.8 | 0 | 0.0 | 0 | 0.0 |
| People who can afford sunscreen | 2 | 1.8 | 1 | 3.3 | 0 | 0.0 | 1 | 3.1 | 0 | 0.0 |
| College educated | 2 | 1.8 | 0 | 0.0 | 0 | 0.0 | 1 | 3.1 | 1 | 3.6 |
| Family / friends | 2 | 1.8 | 0 | 0.0 | 0 | 0.0 | 1 | 3.1 | 1 | 3.6 |
| Native American Population | 1 | 0.9 | 0 | 0.0 | 0 | 0.0 | 0 | 0.0 | 1 | 3.6 |
| People don't care about tan | 1 | 0.9 | 0 | 0.0 | 1 | 4.8 | 0 | 0.0 | 0 | 0.0 |
| City people | 1 | 0.9 | 0 | 0.0 | 0 | 0.0 | 0 | 0.0 | 1 | 3.6 |
| People who are bald | 1 | 0.9 | 0 | 0.0 | 0 | 0.0 | 0 | 0.0 | 1 | 3.6 |
| Scientists / people who believe in science | 1 | 0.9 | 0 | 0.0 | 0 | 0.0 | 0 | 0.0 | 1 | 3.6 |
| People who live in hot states | 1 | 0.9 | 0 | 0.0 | 0 | 0.0 | 0 | 0.0 | 1 | 3.6 |
| **Unlikely to Use** |  |  |  |  |  |  |  |  |  |  |
| Young people | 44 | 39.6 | 17 | 56.7 | 6 | 28.6 | 12 | 37.5 | 9 | 32.1 |
| Men | 17 | 15.3 | 4 | 13.3 | 4 | 19.1 | 7 | 21.9 | 2 | 7.1 |
| People with dark skin | 17 | 15.3 | 4 | 13.3 | 7 | 33.3 | 2 | 6.3 | 4 | 14.3 |
| People who are active outside (e.g. doing work, workouts) | 10 | 9.0 | 2 | 6.7 | 2 | 9.5 | 4 | 12.5 | 2 | 7.1 |
| Older people | 10 | 9.0 | 3 | 10.0 | 1 | 4.8 | 5 | 15.6 | 1 | 3.6 |
| People who don't care about skin health | 7 | 6.3 | 1 | 3.3 | 2 | 9.5 | 3 | 9.4 | 1 | 3.6 |
| People who are too busy / did not plan ahead | 6 | 5.4 | 1 | 3.3 | 0 | 0.0 | 3 | 9.4 | 2 | 7.1 |
| Don't know | 5 | 4.5 | 1 | 3.3 | 0 | 0.0 | 2 | 6.3 | 2 | 7.1 |
| People that do not think they will get sunburn | 5 | 4.5 | 0 | 0.0 | 2 | 9.5 | 3 | 9.4 | 0 | 0.0 |
| People who cannot afford sunscreen / low income | 4 | 3.6 | 2 | 6.7 | 0 | 0.0 | 2 | 6.3 | 0 | 0.0 |
| People who want to be tan / in the sun | 4 | 3.6 | 1 | 3.3 | 0 | 0.0 | 1 | 3.1 | 2 | 7.1 |
| People who don't know benefits of using sunscreen | 4 | 3.6 | 1 | 3.3 | 1 | 4.8 | 1 | 3.1 | 1 | 3.6 |
| Trying to go all natural | 3 | 2.7 | 1 | 3.3 | 0 | 0.0 | 1 | 3.1 | 1 | 3.6 |
| Uneducated | 3 | 2.7 | 0 | 0.0 | 0 | 0.0 | 1 | 3.1 | 2 | 7.1 |
| People who forget | 2 | 1.8 | 0 | 0.0 | 0 | 0.0 | 2 | 6.3 | 0 | 0.0 |
| People who have not had skin cancer | 2 | 1.8 | 0 | 0.0 | 0 | 0.0 | 1 | 3.1 | 1 | 3.6 |
| Left sunscreen at home / ran out of sunscreen | 2 | 1.8 | 0 | 0.0 | 0 | 0.0 | 0 | 0.0 | 2 | 7.1 |
| Too lazy to put it on | 2 | 1.8 | 0 | 0.0 | 0 | 0.0 | 1 | 3.1 | 1 | 3.6 |
| No one is unlikely to use | 2 | 1.8 | 2 | 6.7 | 0 | 0.0 | 0 | 0.0 | 0 | 0.0 |
| People who don't go outside | 1 | 0.9 | 0 | 0.0 | 1 | 4.8 | 0 | 0.0 | 0 | 0.0 |
| People only going outside for a short time | 1 | 0.9 | 0 | 0.0 | 1 | 4.8 | 0 | 0.0 | 0 | 0.0 |
| Most people | 1 | 0.9 | 0 | 0.0 | 0 | 0.0 | 0 | 0.0 | 1 | 3.6 |
| Immigrants | 1 | 0.9 | 0 | 0.0 | 0 | 0.0 | 0 | 0.0 | 1 | 3.6 |
| Religious fanatics | 1 | 0.9 | 0 | 0.0 | 0 | 0.0 | 0 | 0.0 | 1 | 3.6 |
| People who live in the country / rural | 1 | 0.9 | 0 | 0.0 | 0 | 0.0 | 0 | 0.0 | 1 | 3.6 |
| People who wear hats / long sleeves / clothing | 1 | 0.9 | 0 | 0.0 | 0 | 0.0 | 1 | 3.1 | 0 | 0.0 |
| Blue collar males | 1 | 0.9 | 0 | 0.0 | 0 | 0.0 | 0 | 0.0 | 1 | 3.6 |
| Women concerned with body image | 1 | 0.9 | 0 | 0.0 | 1 | 4.8 | 0 | 0.0 | 0 | 0.0 |
| People who want to be tough | 1 | 0.9 | 0 | 0.0 | 0 | 0.0 | 0 | 0.0 | 1 | 3.6 |
| People who live in colder states | 1 | 0.9 | 0 | 0.0 | 0 | 0.0 | 0 | 0.0 | 1 | 3.6 |
| Office workers | 1 | 0.9 | 0 | 0.0 | 1 | 4.8 | 0 | 0.0 | 0 | 0.0 |
| ***Control*** |  |  |  |  |  |  |  |  |  |  |
| **Facilitators / Easier to Use** |  |  |  |  |  |  |  |  |  |  |
| Easily accessible | 25 | 22.5 | 8 | 26.7 | 1 | 4.8 | 7 | 21.9 | 9 | 32.1 |
| Type sunscreen available / spray / mineral sunscreen | 24 | 21.6 | 11 | 36.7 | 3 | 14.3 | 10 | 31.3 | 0 | 0.0 |
| Easier to apply | 18 | 16.2 | 3 | 10.0 | 8 | 38.1 | 5 | 15.6 | 2 | 7.1 |
| Not greasy / sticky | 17 | 15.3 | 2 | 6.7 | 2 | 9.5 | 10 | 31.3 | 3 | 10.7 |
| Cheaper / free | 14 | 12.6 | 9 | 30.0 | 0 | 0.0 | 5 | 15.6 | 0 | 0.0 |
| No additional facilitators needed | 13 | 11.7 | 3 | 10.0 | 2 | 9.5 | 2 | 6.3 | 6 | 21.4 |
| Bring with you | 8 | 7.2 | 3 | 10.0 | 1 | 4.8 | 3 | 9.4 | 1 | 3.6 |
| Longer lasting products | 7 | 6.3 | 2 | 6.7 | 1 | 4.8 | 3 | 9.4 | 1 | 3.6 |
| Unscented or smells good | 6 | 5.4 | 0 | 0.0 | 1 | 4.8 | 3 | 9.4 | 2 | 7.1 |
| Reminders to apply / remembering | 5 | 4.5 | 1 | 3.3 | 3 | 14.3 | 0 | 0.0 | 1 | 3.6 |
| More information about chemicals / ingredients | 5 | 4.5 | 1 | 3.3 | 1 | 4.8 | 2 | 6.3 | 1 | 3.6 |
| Make habitual / part of routine | 4 | 3.6 | 1 | 3.3 | 1 | 4.8 | 1 | 3.1 | 1 | 3.6 |
| Moisturizing skin | 4 | 3.6 | 1 | 3.3 | 0 | 0.0 | 3 | 9.4 | 0 | 0.0 |
| Convenient size | 4 | 3.6 | 0 | 0.0 | 0 | 0.0 | 2 | 6.3 | 2 | 7.1 |
| Know it will prevent sunburn | 3 | 2.7 | 1 | 3.3 | 1 | 4.8 | 0 | 0.0 | 1 | 3.6 |
| Plan to be outside for an extended period | 3 | 2.7 | 0 | 0.0 | 0 | 0.0 | 2 | 6.3 | 1 | 3.6 |
| Someone to put it on | 3 | 2.7 | 1 | 3.3 | 1 | 4.8 | 1 | 3.1 | 0 | 0.0 |
| Previous sunburns | 2 | 1.8 | 0 | 0.0 | 1 | 4.8 | 0 | 0.0 | 1 | 3.6 |
| Lightweight | 2 | 1.8 | 0 | 0.0 | 0 | 0.0 | 2 | 6.3 | 0 | 0.0 |
| Not stain clothing | 2 | 1.8 | 0 | 0.0 | 0 | 0.0 | 1 | 3.1 | 1 | 3.6 |
| Sunscreen that doesn't expire | 2 | 1.8 | 0 | 0.0 | 1 | 4.8 | 0 | 0.0 | 1 | 3.6 |
| Not sweating / exercising | 2 | 1.8 | 0 | 0.0 | 1 | 4.8 | 0 | 0.0 | 1 | 3.6 |
| More time / less busy | 1 | 0.9 | 0 | 0.0 | 1 | 4.8 | 0 | 0.0 | 0 | 0.0 |
| Won't irritate skin or eyes | 1 | 0.9 | 0 | 0.0 | 0 | 0.0 | 1 | 3.1 | 0 | 0.0 |
| Near water | 1 | 0.9 | 1 | 3.3 | 0 | 0.0 | 0 | 0.0 | 0 | 0.0 |
| Less damaging to environment | 1 | 0.9 | 0 | 0.0 | 0 | 0.0 | 0 | 0.0 | 1 | 3.6 |
| Society preferred pale skin | 1 | 0.9 | 0 | 0.0 | 0 | 0.0 | 1 | 3.1 | 0 | 0.0 |
| At high risk of skin cancer | 1 | 0.9 | 0 | 0.0 | 0 | 0.0 | 1 | 3.1 | 0 | 0.0 |
| No wait time before going outside | 1 | 0.9 | 0 | 0.0 | 0 | 0.0 | 1 | 3.1 | 0 | 0.0 |
| Less options / only effective ones available | 1 | 0.9 | 1 | 3.3 | 0 | 0.0 | 0 | 0.0 | 0 | 0.0 |
| Doctor advising | 1 | 0.9 | 0 | 0.0 | 0 | 0.0 | 1 | 3.1 | 0 | 0.0 |
| Right amount | 1 | 0.9 | 0 | 0.0 | 1 | 4.8 | 0 | 0.0 | 0 | 0.0 |
| **Barriers / Harder to Use** |  |  |  |  |  |  |  |  |  |  |
| Not having access / own / near by | 28 | 25.2 | 5 | 16.7 | 6 | 28.6 | 7 | 21.9 | 10 | 35.7 |
| Forgetting | 20 | 18.0 | 7 | 23.3 | 4 | 19.1 | 5 | 15.6 | 4 | 14.3 |
| No barriers | 13 | 11.7 | 5 | 16.7 | 2 | 9.5 | 2 | 6.3 | 4 | 14.3 |
| Being in a hurry / too busy / time consuming | 12 | 10.8 | 3 | 10.0 | 4 | 19.1 | 4 | 12.5 | 1 | 3.6 |
| Greasy / sticky | 8 | 7.2 | 3 | 10.0 | 2 | 9.5 | 2 | 6.3 | 1 | 3.6 |
| Cost | 8 | 7.2 | 4 | 13.3 | 2 | 9.5 | 0 | 0.0 | 2 | 7.1 |
| Active / exercising / sweating | 7 | 6.3 | 1 | 3.3 | 1 | 4.8 | 3 | 9.4 | 2 | 7.1 |
| Wears off / have to reapply | 7 | 6.3 | 2 | 6.7 | 2 | 9.5 | 2 | 6.3 | 1 | 3.6 |
| Hard to apply / apply evenly | 6 | 5.4 | 2 | 6.7 | 1 | 4.8 | 2 | 6.3 | 1 | 3.6 |
| Not being able to reach all areas / being alone | 6 | 5.4 | 1 | 3.3 | 3 | 14.3 | 2 | 6.3 | 0 | 0.0 |
| Not having gluten free sunscreen | 5 | 4.5 | 2 | 6.7 | 1 | 4.8 | 2 | 6.3 | 0 | 0.0 |
| Didn't plan to be outside / outside for long | 4 | 3.6 | 1 | 3.3 | 0 | 0.0 | 2 | 6.3 | 1 | 3.6 |
| Irritates eyes / skin | 4 | 3.6 | 2 | 6.7 | 0 | 0.0 | 2 | 6.3 | 0 | 0.0 |
| Smell | 4 | 3.6 | 0 | 0.0 | 0 | 0.0 | 4 | 12.5 | 0 | 0.0 |
| Sensitive skin | 3 | 2.7 | 1 | 3.3 | 0 | 0.0 | 2 | 6.3 | 0 | 0.0 |
| Not by water to wash hands | 3 | 2.7 | 1 | 3.3 | 0 | 0.0 | 1 | 3.1 | 1 | 3.6 |
| Not knowing what weather will be | 2 | 1.8 | 0 | 0.0 | 1 | 4.8 | 1 | 3.1 | 0 | 0.0 |
| Need to stay clean / don't have time to shower / need to protect clothes | 2 | 1.8 | 1 | 3.3 | 0 | 0.0 | 1 | 3.1 | 0 | 0.0 |
| Being in the water | 2 | 1.8 | 2 | 6.7 | 0 | 0.0 | 0 | 0.0 | 0 | 0.0 |
| Windy / spray hard to use | 2 | 1.8 | 0 | 0.0 | 0 | 0.0 | 2 | 6.3 | 0 | 0.0 |
| Not knowing ingredients / chemicals | 2 | 1.8 | 0 | 0.0 | 1 | 4.8 | 1 | 3.1 | 0 | 0.0 |
| Hot/humid | 2 | 1.8 | 1 | 3.3 | 0 | 0.0 | 1 | 3.1 | 0 | 0.0 |
| Dirt/sand/grass sticking to skin | 2 | 1.8 | 1 | 3.3 | 0 | 0.0 | 1 | 3.1 | 0 | 0.0 |
| It’s a choice | 1 | 0.9 | 0 | 0.0 | 0 | 0.0 | 1 | 3.1 | 0 | 0.0 |
| Not sunny outside | 1 | 0.9 | 0 | 0.0 | 0 | 0.0 | 1 | 3.1 | 0 | 0.0 |
| Goes bad sitting in car | 1 | 0.9 | 0 | 0.0 | 1 | 4.8 | 0 | 0.0 | 0 | 0.0 |
| Not available in stores | 1 | 0.9 | 1 | 3.3 | 0 | 0.0 | 0 | 0.0 | 0 | 0.0 |
| Ran out | 1 | 0.9 | 0 | 0.0 | 0 | 0.0 | 1 | 3.1 | 0 | 0.0 |
| Have to carry | 1 | 0.9 | 1 | 3.3 | 0 | 0.0 | 0 | 0.0 | 0 | 0.0 |
| Need to wait to soak in before going outside | 1 | 0.9 | 1 | 3.3 | 0 | 0.0 | 0 | 0.0 | 0 | 0.0 |
| Kids don't want to wear | 1 | 0.9 | 1 | 3.3 | 0 | 0.0 | 0 | 0.0 | 0 | 0.0 |
| Too many brands / options | 1 | 0.9 | 1 | 3.3 | 0 | 0.0 | 0 | 0.0 | 0 | 0.0 |
| Social situation / 'not cool' | 1 | 0.9 | 0 | 0.0 | 0 | 0.0 | 0 | 0.0 | 1 | 3.6 |
| Inconvenient size | 1 | 0.9 | 0 | 0.0 | 0 | 0.0 | 1 | 3.1 | 0 | 0.0 |
