# Supplementary material for: Sun protection behavior beliefs among adults living in rural United States: A qualitative study in Minnesota
Source: PLoS One. 2025 Sep 12;20(9):e0331685. doi: 10.1371/journal.pone.0331685 (PMC12431656; doi:10.1371/journal.pone.0331685)
Supplement: S2 Table — (DOCX) [file pone.0331685.s006.docx]

**S2 Table. All reported beliefs about seeking shade to reduce sun exposure and prevent sunburn on typical sunny day in the summer.**

|  | **Overall**  **N=96** | | **18-39 Female**  **N=22** | | **18-39 Male**  **N=17** | | **40-60 Female**  **N=34** | | **40-60 Male**  **N=23** | |
| --- | --- | --- | --- | --- | --- | --- | --- | --- | --- | --- |
| **Beliefs** | **n** | **%** | **n** | **%** | **n** | **%** | **n** | **%** | **n** | **%** |
| ***Outcome*** |  |  |  |  |  |  |  |  |  |  |
| **Good/Positive** |  |  |  |  |  |  |  |  |  |  |
| Less cancer / exposure / sunburn | 82 | 85.4 | 21 | 95.5 | 15 | 88.2 | 29 | 85.3 | 17 | 73.9 |
| Protection from heat / stay cool | 73 | 76.0 | 17 | 77.3 | 15 | 88.2 | 27 | 79.4 | 14 | 60.9 |
| Feel comfortable | 19 | 19.8 | 3 | 13.6 | 7 | 41.2 | 2 | 5.9 | 7 | 30.4 |
| Be outside without sun exposure / enjoy outdoors | 16 | 16.7 | 5 | 22.7 | 3 | 17.7 | 7 | 20.6 | 1 | 4.4 |
| Prevent aging / less skin damage | 11 | 11.5 | 4 | 18.2 | 2 | 11.8 | 5 | 14.7 | 0 | 0.0 |
| Less eye strain | 9 | 9.4 | 2 | 9.1 | 1 | 5.9 | 4 | 11.8 | 2 | 8.7 |
| Easier to read / use electronic devices | 8 | 8.3 | 0 | 0.0 | 2 | 11.8 | 6 | 17.7 | 0 | 0.0 |
| Relaxing | 7 | 7.3 | 1 | 4.6 | 1 | 5.9 | 3 | 8.8 | 2 | 8.7 |
| Less sweating | 7 | 7.3 | 2 | 9.1 | 1 | 5.9 | 1 | 2.9 | 3 | 13.0 |
| Lower risk dehydration | 6 | 6.3 | 2 | 9.1 | 1 | 5.9 | 2 | 5.9 | 1 | 4.4 |
| Feel less tired / fatigued | 6 | 6.3 | 1 | 4.6 | 2 | 11.8 | 2 | 5.9 | 1 | 4.4 |
| Avoid using sunscreen | 3 | 3.1 | 2 | 9.1 | 0 | 0.0 | 0 | 0.0 | 1 | 4.4 |
| Break/nap | 2 | 2.1 | 0 | 0.0 | 2 | 11.8 | 0 | 0.0 | 0 | 0.0 |
| No itchy skin / reduce change of skin irritation | 2 | 2.1 | 0 | 0.0 | 1 | 5.9 | 1 | 2.9 | 0 | 0.0 |
| Time to put on sunscreen / reapply | 2 | 2.1 | 1 | 4.6 | 0 | 0.0 | 1 | 2.9 | 0 | 0.0 |
| Less cranky | 1 | 1.0 | 0 | 0.0 | 0 | 0.0 | 1 | 2.9 | 0 | 0.0 |
| "Look smart" | 1 | 1.0 | 0 | 0.0 | 0 | 0.0 | 0 | 0.0 | 1 | 4.4 |
| **Bad/Negative** |  |  |  |  |  |  |  |  |  |  |
| Nothing negative about seeking shade | 32 | 33.3 | 10 | 45.5 | 6 | 35.3 | 9 | 26.5 | 7 | 30.4 |
| No suntan / pale skin | 28 | 29.2 | 9 | 40.9 | 5 | 29.4 | 9 | 26.5 | 5 | 21.7 |
| Away from others / activity / social isolation | 20 | 20.8 | 5 | 22.7 | 4 | 23.5 | 5 | 14.7 | 6 | 26.1 |
| Less vitamin D | 17 | 17.7 | 5 | 22.7 | 4 | 23.5 | 6 | 17.7 | 2 | 8.7 |
| Bugs/Insects | 15 | 15.6 | 4 | 18.2 | 5 | 29.4 | 3 | 8.8 | 3 | 13.0 |
| Too cool (miss warmth of sun) | 14 | 14.6 | 6 | 27.3 | 3 | 17.7 | 4 | 11.8 | 1 | 4.4 |
| Lower work productivity / work not in the shade | 12 | 12.5 | 0 | 0.0 | 4 | 23.5 | 2 | 5.9 | 6 | 26.1 |
| Not always available / need to plan ahead | 11 | 11.5 | 2 | 9.1 | 3 | 17.7 | 4 | 11.8 | 2 | 8.7 |
| Sunlight improves mental health / feels good | 7 | 7.3 | 3 | 13.6 | 0 | 0.0 | 4 | 11.8 | 0 | 0.0 |
| Harder to exercise / less mobile | 6 | 6.3 | 0 | 0.0 | 1 | 5.9 | 3 | 8.8 | 2 | 8.7 |
| Harder at beach / pool / lake | 4 | 4.2 | 0 | 0.0 | 0 | 0.0 | 4 | 11.8 | 0 | 0.0 |
| Limited space | 4 | 4.2 | 1 | 4.6 | 0 | 0.0 | 1 | 2.9 | 2 | 8.7 |
| Social stigma | 3 | 3.1 | 1 | 4.6 | 1 | 5.9 | 1 | 2.9 | 0 | 0.0 |
| Darker / hard to see in shade / may block view | 2 | 2.1 | 0 | 0.0 | 1 | 5.9 | 1 | 2.9 | 0 | 0.0 |
| Arm sore / issues using umbrella | 2 | 2.1 | 1 | 4.6 | 0 | 0.0 | 1 | 2.9 | 0 | 0.0 |
| Fear of future sunburn due to less sun exposure | 2 | 2.1 | 1 | 4.6 | 0 | 0.0 | 0 | 0.0 | 1 | 4.4 |
| Less sun exposure | 2 | 2.1 | 0 | 0.0 | 0 | 0.0 | 2 | 5.9 | 0 | 0.0 |
| May not fully prevent sun exposure / sunburn | 2 | 2.1 | 0 | 0.0 | 0 | 0.0 | 1 | 2.9 | 1 | 4.4 |
| Fall asleep / get too comfortable | 2 | 2.1 | 0 | 0.0 | 1 | 5.9 | 0 | 0.0 | 1 | 4.4 |
| ***Normative*** |  |  |  |  |  |  |  |  |  |  |
| **Approve/Support Use** |  |  |  |  |  |  |  |  |  |  |
| Friends / family | 27 | 28.1 | 5 | 22.7 | 4 | 23.5 | 9 | 26.5 | 9 | 39.1 |
| Most people / everyone | 22 | 22.9 | 9 | 40.9 | 4 | 23.5 | 8 | 23.5 | 1 | 4.4 |
| Healthcare providers | 20 | 20.8 | 6 | 27.3 | 4 | 23.5 | 6 | 17.7 | 4 | 17.4 |
| Older people | 18 | 18.8 | 2 | 9.1 | 3 | 17.7 | 10 | 29.4 | 3 | 13.0 |
| People with personal or family history of skin cancer | 9 | 9.4 | 3 | 13.6 | 2 | 11.8 | 2 | 5.9 | 2 | 8.7 |
| People with children | 5 | 5.2 | 0 | 0.0 | 0 | 0.0 | 4 | 11.8 | 1 | 4.4 |
| Fair skin / reactive to sun exposure | 5 | 5.2 | 0 | 0.0 | 1 | 5.9 | 2 | 5.9 | 2 | 8.7 |
| Smart people | 4 | 4.2 | 0 | 0.0 | 0 | 0.0 | 1 | 2.9 | 3 | 13.0 |
| Do not like being in sun | 3 | 3.1 | 1 | 4.6 | 0 | 0.0 | 1 | 2.9 | 1 | 4.4 |
| People who get hot / sweaty | 3 | 3.1 | 0 | 0.0 | 1 | 5.9 | 2 | 5.9 | 0 | 0.0 |
| Outdoor enthusiasts / spend a lot of time outdoors | 3 | 3.1 | 1 | 4.6 | 0 | 0.0 | 2 | 5.9 | 0 | 0.0 |
| Young children | 3 | 3.1 | 0 | 0.0 | 0 | 0.0 | 2 | 5.9 | 1 | 4.4 |
| Don't care / don't need approval from others | 3 | 3.1 | 1 | 4.6 | 1 | 5.9 | 1 | 2.9 | 0 | 0.0 |
| Adults | 3 | 3.1 | 1 | 4.6 | 1 | 5.9 | 0 | 0.0 | 1 | 4.4 |
| Employer | 2 | 2.1 | 0 | 0.0 | 2 | 11.8 | 0 | 0.0 | 0 | 0.0 |
| No one approves | 1 | 1.0 | 0 | 0.0 | 1 | 5.9 | 0 | 0.0 | 0 | 0.0 |
| Overweight people | 1 | 1.0 | 0 | 0.0 | 1 | 5.9 | 0 | 0.0 | 0 | 0.0 |
| Lifeguards | 1 | 1.0 | 0 | 0.0 | 1 | 5.9 | 0 | 0.0 | 0 | 0.0 |
| People who worry a lot | 1 | 1.0 | 0 | 0.0 | 0 | 0.0 | 0 | 0.0 | 1 | 4.4 |
| Men | 1 | 1.0 | 0 | 0.0 | 1 | 5.9 | 0 | 0.0 | 0 | 0.0 |
| Understand skin cancer risk | 1 | 1.0 | 0 | 0.0 | 0 | 0.0 | 1 | 2.9 | 0 | 0.0 |
| Tan people | 1 | 1.0 | 0 | 0.0 | 0 | 0.0 | 0 | 0.0 | 1 | 4.4 |
| **Disapprove/Not Support Use** |  |  |  |  |  |  |  |  |  |  |
| No one disapproves | 30 | 31.3 | 10 | 45.5 | 6 | 35.3 | 11 | 32.4 | 3 | 13.0 |
| Young people | 13 | 13.5 | 0 | 0.0 | 3 | 17.7 | 7 | 20.6 | 3 | 13.0 |
| People who want to be tan / in the sun | 12 | 12.5 | 2 | 9.1 | 1 | 5.9 | 7 | 20.6 | 2 | 8.7 |
| Employers | 10 | 10.4 | 1 | 4.6 | 3 | 17.7 | 3 | 8.8 | 3 | 13.0 |
| People who are active | 7 | 7.3 | 1 | 4.6 | 1 | 5.9 | 2 | 5.9 | 3 | 13.0 |
| Coworkers / teammates / partner | 6 | 6.3 | 1 | 4.6 | 1 | 5.9 | 1 | 2.9 | 3 | 13.0 |
| Don't care what other people think | 6 | 6.3 | 2 | 9.1 | 0 | 0.0 | 3 | 8.8 | 1 | 4.4 |
| Don't know | 5 | 5.2 | 2 | 9.1 | 1 | 5.9 | 2 | 5.9 | 0 | 0.0 |
| Companies that produce other sun protection (sunscreen, clothing) | 3 | 3.1 | 1 | 4.6 | 1 | 5.9 | 0 | 0.0 | 1 | 4.4 |
| Don't understand sun exposure and skin cancer risk | 3 | 3.1 | 1 | 4.6 | 1 | 5.9 | 1 | 2.9 | 0 | 0.0 |
| Friends / family | 2 | 2.1 | 0 | 0.0 | 2 | 11.8 | 0 | 0.0 | 0 | 0.0 |
| People with dark skin | 2 | 2.1 | 0 | 0.0 | 0 | 0.0 | 1 | 2.9 | 1 | 4.4 |
| Stupid people | 1 | 1.0 | 0 | 0.0 | 0 | 0.0 | 0 | 0.0 | 1 | 4.4 |
| Healthcare providers | 1 | 1.0 | 1 | 4.6 | 0 | 0.0 | 0 | 0.0 | 0 | 0.0 |
| People with pale skin | 1 | 1.0 | 0 | 0.0 | 0 | 0.0 | 0 | 0.0 | 1 | 4.4 |
| People already in shady spot / people who don't like sharing shade | 1 | 1.0 | 1 | 4.6 | 0 | 0.0 | 0 | 0.0 | 0 | 0.0 |
| Illegal day laborers | 1 | 1.0 | 0 | 0.0 | 1 | 5.9 | 0 | 0.0 | 0 | 0.0 |
| Myself | 1 | 1.0 | 0 | 0.0 | 0 | 0.0 | 0 | 0.0 | 1 | 4.4 |
| Tough guys | 1 | 1.0 | 0 | 0.0 | 1 | 5.9 | 0 | 0.0 | 0 | 0.0 |
| **Likely to Use** |  |  |  |  |  |  |  |  |  |  |
| Older people | 48 | 50.0 | 11 | 50.0 | 7 | 41.2 | 19 | 55.9 | 11 | 47.8 |
| People with fair skin / prone to sunburn | 28 | 29.2 | 9 | 40.9 | 5 | 29.4 | 9 | 26.5 | 5 | 21.7 |
| People with personal or family history of skin cancer | 17 | 17.7 | 8 | 36.4 | 3 | 17.7 | 5 | 14.7 | 1 | 4.4 |
| People who get hot / sweaty | 12 | 12.5 | 6 | 27.3 | 1 | 5.9 | 3 | 8.8 | 2 | 8.7 |
| Parents/people with children | 12 | 12.5 | 4 | 18.2 | 1 | 5.9 | 5 | 14.7 | 2 | 8.7 |
| Children / babies | 8 | 8.3 | 1 | 4.6 | 1 | 5.9 | 4 | 11.8 | 2 | 8.7 |
| Knowledge of sun exposure consequences | 7 | 7.3 | 2 | 9.1 | 1 | 5.9 | 3 | 8.8 | 1 | 4.4 |
| Health conscious | 7 | 7.3 | 0 | 0.0 | 1 | 5.9 | 4 | 11.8 | 2 | 8.7 |
| People without sun protective clothing or sunscreen | 3 | 3.1 | 0 | 0.0 | 1 | 5.9 | 1 | 2.9 | 1 | 4.4 |
| Outdoor enthusiasts | 3 | 3.1 | 0 | 0.0 | 1 | 5.9 | 1 | 2.9 | 1 | 4.4 |
| Most people | 3 | 3.1 | 0 | 0.0 | 2 | 11.8 | 1 | 2.9 | 0 | 0.0 |
| Those on medications that indicate limited sun exposure | 3 | 3.1 | 0 | 0.0 | 1 | 5.9 | 2 | 5.9 | 0 | 0.0 |
| People who work outdoors | 3 | 3.1 | 0 | 0.0 | 2 | 11.8 | 1 | 2.9 | 0 | 0.0 |
| People who are overweight | 3 | 3.1 | 1 | 4.6 | 1 | 5.9 | 1 | 2.9 | 0 | 0.0 |
| Lazy people / don't like to work | 3 | 3.1 | 0 | 0.0 | 0 | 0.0 | 0 | 0.0 | 3 | 13.0 |
| Friends / family | 3 | 3.1 | 1 | 4.6 | 1 | 5.9 | 0 | 0.0 | 1 | 4.4 |
| Healthcare providers | 3 | 3.1 | 2 | 9.1 | 0 | 0.0 | 1 | 2.9 | 0 | 0.0 |
| People with pets | 2 | 2.1 | 1 | 4.6 | 0 | 0.0 | 1 | 2.9 | 0 | 0.0 |
| Women | 2 | 2.1 | 1 | 4.6 | 0 | 0.0 | 0 | 0.0 | 1 | 4.4 |
| People who don't work outside | 2 | 2.1 | 0 | 0.0 | 0 | 0.0 | 0 | 0.0 | 2 | 8.7 |
| Smart people | 2 | 2.1 | 0 | 0.0 | 0 | 0.0 | 0 | 0.0 | 0 | 0.0 |
| Those with lots of trees in their area | 1 | 1.0 | 1 | 4.6 | 0 | 0.0 | 0 | 0.0 | 0 | 0.0 |
| Me | 1 | 1.0 | 1 | 4.6 | 0 | 0.0 | 0 | 0.0 | 0 | 0.0 |
| People with dark skin | 1 | 1.0 | 0 | 0.0 | 0 | 0.0 | 1 | 2.9 | 0 | 0.0 |
| People who plan ahead | 1 | 1.0 | 0 | 0.0 | 0 | 0.0 | 0 | 0.0 | 1 | 4.4 |
| Vacationers | 1 | 1.0 | 0 | 0.0 | 0 | 0.0 | 0 | 0.0 | 1 | 4.4 |
| Homeless | 1 | 1.0 | 0 | 0.0 | 1 | 5.9 | 0 | 0.0 | 0 | 0.0 |
| City folk | 1 | 1.0 | 0 | 0.0 | 1 | 5.9 | 0 | 0.0 | 0 | 0.0 |
| People who go to church | 1 | 1.0 | 0 | 0.0 | 1 | 5.9 | 0 | 0.0 | 0 | 0.0 |
| Those who prefer the shade | 1 | 1.0 | 0 | 0.0 | 0 | 0.0 | 1 | 2.9 | 0 | 0.0 |
| **Unlikely to Use** |  |  |  |  |  |  |  |  |  |  |
| Young people | 47 | 49.0 | 11 | 50.0 | 7 | 41.2 | 21 | 61.8 | 8 | 34.8 |
| People who want to be tan / in the sun | 20 | 20.8 | 6 | 27.3 | 3 | 17.7 | 8 | 23.5 | 3 | 13.0 |
| People who are active outside (e.g., doing work / workouts) | 17 | 17.7 | 2 | 9.1 | 2 | 11.8 | 3 | 8.8 | 10 | 43.5 |
| People with darker skin / do not burn easily | 8 | 8.3 | 1 | 4.6 | 3 | 17.7 | 3 | 8.8 | 1 | 4.4 |
| People attending outdoor events | 7 | 7.3 | 2 | 9.1 | 0 | 0.0 | 2 | 5.9 | 3 | 13.0 |
| Not concerned about sun exposure risks / don't pay attention to time spent outside | 6 | 6.3 | 1 | 4.6 | 1 | 5.9 | 4 | 11.8 | 0 | 0.0 |
| Older people | 5 | 5.2 | 0 | 0.0 | 0 | 0.0 | 2 | 5.9 | 3 | 13.0 |
| People doing water activities | 4 | 4.2 | 1 | 4.6 | 0 | 0.0 | 2 | 5.9 | 1 | 4.4 |
| People who like to be hot / sensitive to cold | 4 | 4.2 | 1 | 4.6 | 1 | 5.9 | 1 | 2.9 | 1 | 4.4 |
| Unaware of sun exposure risks | 3 | 3.1 | 0 | 0.0 | 1 | 5.9 | 2 | 5.9 | 0 | 0.0 |
| No one is unlikely to use | 2 | 2.1 | 0 | 0.0 | 1 | 5.9 | 0 | 0.0 | 1 | 4.4 |
| Uneducated people | 2 | 2.1 | 1 | 4.6 | 1 | 5.9 | 0 | 0.0 | 0 | 0.0 |
| People who have integrity / good work ethic | 2 | 2.1 | 0 | 0.0 | 0 | 0.0 | 0 | 0.0 | 2 | 8.7 |
| Men | 2 | 2.1 | 0 | 0.0 | 1 | 5.9 | 0 | 0.0 | 1 | 4.4 |
| People with sun protective clothing | 2 | 2.1 | 0 | 0.0 | 1 | 5.9 | 0 | 0.0 | 1 | 4.4 |
| No trees in area | 1 | 1.0 | 1 | 4.6 | 0 | 0.0 | 0 | 0.0 | 0 | 0.0 |
| People not affected by skin cancer | 1 | 1.0 | 0 | 0.0 | 0 | 0.0 | 1 | 2.9 | 0 | 0.0 |
| People who work inside all day | 1 | 1.0 | 0 | 0.0 | 0 | 0.0 | 1 | 2.9 | 0 | 0.0 |
| People who fear judgement from others | 1 | 1.0 | 0 | 0.0 | 0 | 0.0 | 1 | 2.9 | 0 | 0.0 |
| Women | 1 | 1.0 | 0 | 0.0 | 1 | 5.9 | 0 | 0.0 | 0 | 0.0 |
| Risky people | 1 | 1.0 | 0 | 0.0 | 0 | 0.0 | 0 | 0.0 | 1 | 4.4 |
| People living in northern climates | 1 | 1.0 | 1 | 4.6 | 0 | 0.0 | 0 | 0.0 | 0 | 0.0 |
| Foreign workers | 1 | 1.0 | 0 | 0.0 | 1 | 5.9 | 0 | 0.0 | 0 | 0.0 |
| ***Control*** |  |  |  |  |  |  |  |  |  |  |
| **Facilitators / Easier to Use** |  |  |  |  |  |  |  |  |  |  |
| Availability of shaded areas | 31 | 32.3 | 7 | 31.8 | 5 | 29.4 | 12 | 35.3 | 7 | 30.4 |
| Availability of trees | 19 | 19.8 | 4 | 18.2 | 1 | 5.9 | 7 | 20.6 | 7 | 30.4 |
| Canopy / pavilion / umbrella available at location | 17 | 17.7 | 1 | 4.6 | 0 | 0.0 | 11 | 32.4 | 5 | 21.7 |
| Really hot / humid | 16 | 16.7 | 7 | 31.8 | 3 | 17.7 | 4 | 11.8 | 2 | 8.7 |
| Wanting to read / do activity in shade / something to do in shade | 9 | 9.4 | 1 | 4.6 | 3 | 17.7 | 4 | 11.8 | 1 | 4.4 |
| Bring umbrella / equipment / hat | 9 | 9.4 | 2 | 9.1 | 0 | 0.0 | 4 | 11.8 | 3 | 13.0 |
| Place to sit in shade | 6 | 6.3 | 0 | 0.0 | 1 | 5.9 | 5 | 14.7 | 0 | 0.0 |
| Not doing an activity / exercising | 5 | 5.2 | 0 | 0.0 | 2 | 11.8 | 2 | 5.9 | 1 | 4.4 |
| Other people in the shade | 4 | 4.2 | 0 | 0.0 | 1 | 5.9 | 1 | 2.9 | 2 | 8.7 |
| Very sunny / high UV index / few clouds | 4 | 4.2 | 2 | 9.1 | 1 | 5.9 | 1 | 2.9 | 0 | 0.0 |
| Knowledge about danger of sun exposure | 3 | 3.1 | 1 | 4.6 | 1 | 5.9 | 1 | 2.9 | 0 | 0.0 |
| Someone else do the work needed / not having work outside | 3 | 3.1 | 0 | 0.0 | 1 | 5.9 | 0 | 0.0 | 2 | 8.7 |
| Bug spray applied | 3 | 3.1 | 0 | 0.0 | 0 | 0.0 | 2 | 5.9 | 1 | 4.4 |
| Plan ahead / be proactive | 3 | 3.1 | 1 | 4.6 | 1 | 5.9 | 1 | 2.9 | 0 | 0.0 |
| Near activity / distance to main activity | 2 | 2.1 | 0 | 0.0 | 0 | 0.0 | 0 | 0.0 | 2 | 8.7 |
| Water to drink nearby / need to rehydrate | 2 | 2.1 | 0 | 0.0 | 0 | 0.0 | 1 | 2.9 | 1 | 4.4 |
| No sunscreen on / available | 2 | 2.1 | 1 | 4.6 | 1 | 5.9 | 0 | 0.0 | 0 | 0.0 |
| Plan to be outside for long period | 2 | 2.1 | 0 | 0.0 | 1 | 5.9 | 1 | 2.9 | 0 | 0.0 |
| Other needs / prefer shade (e.g. child) | 2 | 2.1 | 2 | 9.1 | 0 | 0.0 | 0 | 0.0 | 0 | 0.0 |
| Breeze | 2 | 2.1 | 0 | 0.0 | 0 | 0.0 | 2 | 5.9 | 0 | 0.0 |
| Forgot sun hat | 1 | 1.0 | 0 | 0.0 | 1 | 5.9 | 0 | 0.0 | 0 | 0.0 |
| Prolonged sun exposure / already sunburned | 1 | 1.0 | 1 | 4.6 | 0 | 0.0 | 0 | 0.0 | 0 | 0.0 |
| Time to assemble canopy / create shade | 1 | 1.0 | 0 | 0.0 | 1 | 5.9 | 0 | 0.0 | 0 | 0.0 |
| Not having a lake home | 1 | 1.0 | 0 | 0.0 | 1 | 5.9 | 0 | 0.0 | 0 | 0.0 |
| Affordable shade equipment | 1 | 1.0 | 1 | 4.6 | 0 | 0.0 | 0 | 0.0 | 0 | 0.0 |
| Staying in the same place | 1 | 1.0 | 1 | 4.6 | 0 | 0.0 | 0 | 0.0 | 0 | 0.0 |
| Skin condition | 1 | 1.0 | 1 | 4.6 | 0 | 0.0 | 0 | 0.0 | 0 | 0.0 |
| **Barriers / Harder to Use** |  |  |  |  |  |  |  |  |  |  |
| No shade available | 44 | 45.8 | 12 | 54.6 | 3 | 17.7 | 19 | 55.9 | 10 | 43.5 |
| Job / work requires sun exposure | 20 | 20.8 | 2 | 9.1 | 7 | 41.2 | 3 | 8.8 | 8 | 34.8 |
| No/not many trees | 17 | 17.7 | 5 | 22.7 | 3 | 17.7 | 4 | 11.8 | 5 | 21.7 |
| Participating in sports / activity where shade isn't possible / being active | 17 | 17.7 | 3 | 13.6 | 2 | 11.8 | 10 | 29.4 | 2 | 8.7 |
| Temperature too cool | 10 | 10.4 | 5 | 22.7 | 4 | 23.5 | 1 | 2.9 | 0 | 0.0 |
| No umbrella available / forgot | 8 | 8.3 | 4 | 18.2 | 0 | 0.0 | 1 | 2.9 | 3 | 13.0 |
| Socially / others want to be in the sun | 5 | 5.2 | 2 | 9.1 | 0 | 0.0 | 2 | 5.9 | 1 | 4.4 |
| Far from activity / others / nothing to do | 4 | 4.2 | 0 | 0.0 | 1 | 5.9 | 2 | 5.9 | 1 | 4.4 |
| Too many people in shade area | 3 | 3.1 | 1 | 4.6 | 0 | 0.0 | 1 | 2.9 | 1 | 4.4 |
| No equipment to sit in shade | 2 | 2.1 | 0 | 0.0 | 0 | 0.0 | 0 | 0.0 | 0 | 0.0 |
| Time of day | 2 | 2.1 | 0 | 0.0 | 0 | 0.0 | 1 | 2.9 | 1 | 4.4 |
| Short summer season (MN) | 2 | 2.1 | 1 | 4.6 | 0 | 0.0 | 0 | 0.0 | 1 | 4.4 |
| Dirty / uneven ground in shade | 2 | 2.1 | 0 | 0.0 | 0 | 0.0 | 2 | 5.9 | 0 | 0.0 |
| Bugs/insects | 2 | 2.1 | 0 | 0.0 | 0 | 0.0 | 1 | 2.9 | 1 | 4.4 |
| Children | 1 | 1.0 | 1 | 4.6 | 0 | 0.0 | 0 | 0.0 | 0 | 0.0 |
| Having sunscreen | 1 | 1.0 | 1 | 4.6 | 0 | 0.0 | 0 | 0.0 | 0 | 0.0 |
| Wanting a tan | 1 | 1.0 | 1 | 4.6 | 0 | 0.0 | 0 | 0.0 | 0 | 0.0 |
| Time (outside for a short amount of time) | 1 | 1.0 | 0 | 0.0 | 0 | 0.0 | 1 | 2.9 | 0 | 0.0 |
| Cloud cover | 1 | 1.0 | 0 | 0.0 | 1 | 5.9 | 0 | 0.0 | 0 | 0.0 |
| Distracting / forget | 1 | 1.0 | 0 | 0.0 | 0 | 0.0 | 0 | 0.0 | 1 | 4.4 |
| Wearing a hat | 1 | 1.0 | 0 | 0.0 | 1 | 5.9 | 0 | 0.0 | 0 | 0.0 |
| Feel judged | 1 | 1.0 | 1 | 4.6 | 0 | 0.0 | 0 | 0.0 | 0 | 0.0 |
