# Supplementary material for: Sun protection behavior beliefs among adults living in rural United States: A qualitative study in Minnesota
Source: PLoS One. 2025 Sep 12;20(9):e0331685. doi: 10.1371/journal.pone.0331685 (PMC12431656; doi:10.1371/journal.pone.0331685)
Supplement: S3 Table — (DOCX) [file pone.0331685.s007.docx]

**S3 Table. All reported beliefs about wearing clothing to reduce sun exposure and prevent sunburn on typical sunny day in the summer.**

|  | **Overall**  **N=117** | | **18-39 Female**  **N=31** | | **18-39 Male**  **N=27** | | **40-60 Female**  **N=31** | | **40-60 Male**  **N=28** | |
| --- | --- | --- | --- | --- | --- | --- | --- | --- | --- | --- |
| **Beliefs** | **n** | **%** | **n** | **%** | **n** | **%** | **n** | **%** | **n** | **%** |
| ***Behavioral*** |  |  |  |  |  |  |  |  |  |  |
| **Good/Positive** |  |  |  |  |  |  |  |  |  |  |
| Less cancer / exposure / sunburn | 106 | 90.6 | 30 | 96.8 | 22 | 81.5 | 31 | 100.0 | 23 | 82.1 |
| Don't have to apply sunscreen (chemicals, save money, forget) | 25 | 21.4 | 11 | 35.5 | 9 | 33.3 | 4 | 12.9 | 1 | 3.6 |
| Less / prevent bug bites / need for less bug spray | 13 | 11.1 | 4 | 12.9 | 1 | 3.7 | 6 | 19.4 | 2 | 7.1 |
| Protection from heat / stay cool | 12 | 10.3 | 2 | 6.5 | 5 | 18.5 | 2 | 6.5 | 3 | 10.7 |
| Reduce skin peeling / pain / sunburn discomfort | 12 | 10.3 | 1 | 3.2 | 3 | 11.1 | 5 | 16.1 | 3 | 10.7 |
| Prevent aging / less skin damage | 10 | 8.5 | 4 | 12.9 | 1 | 3.7 | 4 | 12.9 | 1 | 3.6 |
| Stay outside longer / can enjoy the day | 6 | 5.1 | 1 | 3.2 | 0 | 0.0 | 3 | 9.7 | 2 | 7.1 |
| Stay warm on windy / cooler days / Protects against wind / wind whip | 5 | 4.3 | 2 | 6.5 | 1 | 3.7 | 0 | 0.0 | 2 | 7.1 |
| Nothing positive about wearing protective clothing | 4 | 3.4 | 1 | 3.2 | 0 | 0.0 | 1 | 3.2 | 2 | 7.1 |
| Lower anxiety / worry about future health problems | 3 | 2.6 | 0 | 0.0 | 0 | 0.0 | 2 | 6.5 | 1 | 3.6 |
| Cover skin don't want exposed (fat, pale skin) | 2 | 1.7 | 1 | 3.2 | 0 | 0.0 | 0 | 0.0 | 1 | 3.6 |
| Skin less dry | 2 | 1.7 | 0 | 0.0 | 0 | 0.0 | 2 | 6.5 | 0 | 0.0 |
| Protection from branches/thorns/tools/equipment | 2 | 1.7 | 0 | 0.0 | 0 | 0.0 | 1 | 3.2 | 1 | 3.6 |
| More activity freedom | 1 | 0.9 | 0 | 0.0 | 0 | 0.0 | 0 | 0.0 | 1 | 3.6 |
| Many good / comfortable options | 1 | 0.9 | 0 | 0.0 | 0 | 0.0 | 1 | 3.2 | 0 | 0.0 |
| Hands free (no umbrella) | 1 | 0.9 | 1 | 3.2 | 0 | 0.0 | 0 | 0.0 | 0 | 0.0 |
| Soak up sweat / less sweaty | 1 | 0.9 | 0 | 0.0 | 0 | 0.0 | 0 | 0.0 | 1 | 3.6 |
| Don't tire as easily / heat | 1 | 0.9 | 0 | 0.0 | 0 | 0.0 | 1 | 3.2 | 0 | 0.0 |
| Taking care of myself | 1 | 0.9 | 0 | 0.0 | 0 | 0.0 | 1 | 3.2 | 0 | 0.0 |
| Reduce fade of tattoos | 1 | 0.9 | 1 | 3.2 | 0 | 0.0 | 0 | 0.0 | 0 | 0.0 |
| More use of winter clothes | 1 | 0.9 | 0 | 0.0 | 0 | 0.0 | 1 | 3.2 | 0 | 0.0 |
| **Bad/Negative** |  |  |  |  |  |  |  |  |  |  |
| Sweat / overheating | 97 | 82.9 | 27 | 87.1 | 22 | 81.5 | 26 | 83.9 | 22 | 78.6 |
| Uncomfortable / skin irritation | 26 | 22.2 | 6 | 19.4 | 8 | 29.6 | 6 | 19.4 | 6 | 21.4 |
| No suntan / pale skin | 22 | 18.8 | 6 | 19.4 | 5 | 18.5 | 8 | 25.8 | 3 | 10.7 |
| Cost / need to buy new clothes | 13 | 11.1 | 5 | 16.1 | 2 | 7.4 | 4 | 12.9 | 2 | 7.1 |
| Nothing negative about wearing protective clothing | 12 | 10.3 | 3 | 9.7 | 2 | 7.4 | 3 | 9.7 | 4 | 14.3 |
| ight / restricted movement during activity | 12 | 10.3 | 3 | 9.7 | 3 | 11.1 | 4 | 12.9 | 2 | 7.1 |
| Tan lines | 8 | 6.8 | 3 | 9.7 | 1 | 3.7 | 3 | 9.7 | 1 | 3.6 |
| Don't look like others / overdressed / comments from others / people starting | 7 | 6.0 | 1 | 3.2 | 1 | 3.7 | 3 | 9.7 | 2 | 7.1 |
| Limited options / not fashionable | 6 | 5.1 | 1 | 3.2 | 1 | 3.7 | 3 | 9.7 | 1 | 3.6 |
| Not enough vitamin D | 6 | 5.1 | 1 | 3.2 | 1 | 3.7 | 1 | 3.2 | 3 | 10.7 |
| Dehydration | 4 | 3.4 | 0 | 0.0 | 1 | 3.7 | 2 | 6.5 | 1 | 3.6 |
| Short MN summer | 4 | 3.4 | 0 | 0.0 | 2 | 7.4 | 2 | 6.5 | 0 | 0.0 |
| Time consuming / inconvenient / switch clothes | 4 | 3.4 | 2 | 6.5 | 0 | 0.0 | 2 | 6.5 | 0 | 0.0 |
| Chafing | 3 | 2.6 | 0 | 0.0 | 2 | 7.4 | 0 | 0.0 | 1 | 3.6 |
| Can't enjoy outdoors / would limit time outside | 3 | 2.6 | 1 | 3.2 | 1 | 3.7 | 1 | 3.2 | 0 | 0.0 |
| Miss warm sun / miss benefits of being in sun | 3 | 2.6 | 1 | 3.2 | 1 | 3.7 | 0 | 0.0 | 1 | 3.6 |
| Being covered up / not showing body | 2 | 1.7 | 1 | 3.2 | 1 | 3.7 | 0 | 0.0 | 0 | 0.0 |
| "still need sunscreen" | 2 | 1.7 | 1 | 3.2 | 0 | 0.0 | 1 | 3.2 | 0 | 0.0 |
| Cancer | 1 | 0.9 | 0 | 0.0 | 1 | 3.7 | 0 | 0.0 | 0 | 0.0 |
| Clothing snagged while working / yard work | 1 | 0.9 | 1 | 3.2 | 0 | 0.0 | 0 | 0.0 | 0 | 0.0 |
| Dry skin | 1 | 0.9 | 0 | 0.0 | 0 | 0.0 | 1 | 3.2 | 0 | 0.0 |
| Forget to do / have to bring | 1 | 0.9 | 0 | 0.0 | 0 | 0.0 | 1 | 3.2 | 0 | 0.0 |
| More laundry | 1 | 0.9 | 0 | 0.0 | 1 | 3.7 | 0 | 0.0 | 0 | 0.0 |
| Tired faster (because of heat) | 1 | 0.9 | 1 | 3.2 | 0 | 0.0 | 0 | 0.0 | 0 | 0.0 |
| ***Normative*** |  |  |  |  |  |  |  |  |  |  |
| **Approve/Support Use** |  |  |  |  |  |  |  |  |  |  |
| Family / Friends | 34 | 29.0 | 13 | 41.9 | 2 | 7.4 | 9 | 29.1 | 10 | 35.7 |
| Healthcare providers | 24 | 20.5 | 6 | 19.4 | 7 | 25.9 | 8 | 25.8 | 3 | 10.7 |
| Older people | 22 | 18.8 | 4 | 12.9 | 6 | 22.2 | 9 | 29.0 | 3 | 10.7 |
| Most people / everyone | 21 | 17.9 | 8 | 25.8 | 5 | 18.5 | 4 | 12.9 | 4 | 14.3 |
| People with personal / family history of skin cancer | 13 | 11.1 | 5 | 16.1 | 2 | 7.4 | 5 | 16.1 | 1 | 3.6 |
| Parents/people with children | 9 | 7.7 | 2 | 6.5 | 4 | 14.8 | 3 | 9.7 | 0 | 0.0 |
| Fair complexion / red hair | 9 | 7.7 | 1 | 3.2 | 4 | 14.8 | 2 | 6.5 | 2 | 7.1 |
| Don't care what other people think | 5 | 4.3 | 0 | 0.0 | 2 | 7.4 | 0 | 0.0 | 3 | 10.7 |
| People who are (physically) active | 5 | 4.3 | 1 | 3.2 | 1 | 3.7 | 3 | 9.7 | 0 | 0.0 |
| Children | 4 | 3.4 | 2 | 6.5 | 0 | 0.0 | 1 | 3.2 | 1 | 3.6 |
| People who know clothing protects against sun / health conscious | 4 | 3.4 | 0 | 0.0 | 1 | 3.7 | 2 | 6.5 | 1 | 3.6 |
| Employer / co-workers | 3 | 2.6 | 1 | 3.2 | 1 | 3.7 | 0 | 0.0 | 1 | 3.6 |
| I don't know | 3 | 2.6 | 2 | 6.5 | 0 | 0.0 | 1 | 3.2 | 0 | 0.0 |
| Smart people / educated people | 3 | 2.6 | 1 | 3.2 | 1 | 3.7 | 0 | 0.0 | 1 | 3.6 |
| Left wing extremists / liberals | 2 | 1.7 | 0 | 0.0 | 1 | 3.7 | 0 | 0.0 | 1 | 3.6 |
| Clothing companies | 2 | 1.7 | 2 | 6.5 | 0 | 0.0 | 0 | 0.0 | 0 | 0.0 |
| People at outdoor all-day event | 1 | 0.9 | 0 | 0.0 | 0 | 0.0 | 0 | 0.0 | 1 | 3.6 |
| Anyone who wants to prevent UV exposure | 1 | 0.9 | 0 | 0.0 | 0 | 0.0 | 1 | 3.2 | 0 | 0.0 |
| People from Middle East | 1 | 0.9 | 1 | 3.2 | 0 | 0.0 | 0 | 0.0 | 0 | 0.0 |
| People that don't work outside | 1 | 0.9 | 0 | 0.0 | 1 | 3.7 | 0 | 0.0 | 0 | 0.0 |
| Those taking medications that make them sensitive to UV | 1 | 0.9 | 1 | 3.2 | 0 | 0.0 | 0 | 0.0 | 0 | 0.0 |
| Skin cancer foundations | 1 | 0.9 | 0 | 0.0 | 0 | 0.0 | 0 | 0.0 | 1 | 3.6 |
| Scientists / environmentalists | 1 | 0.9 | 0 | 0.0 | 0 | 0.0 | 0 | 0.0 | 1 | 3.6 |
| Women | 1 | 0.9 | 0 | 0.0 | 1 | 3.7 | 0 | 0.0 | 0 | 0.0 |
| Asians | 1 | 0.9 | 1 | 3.2 | 0 | 0.0 | 0 | 0.0 | 0 | 0.0 |
| People who have been sunburned | 1 | 0.9 | 0 | 0.0 | 1 | 3.7 | 0 | 0.0 | 0 | 0.0 |
| **Disapprove/Not Support Use** |  |  |  |  |  |  |  |  |  |  |
| No one disapproves | 40 | 34.2 | 12 | 38.7 | 10 | 37.0 | 9 | 29.0 | 9 | 32.1 |
| Young people | 25 | 21.4 | 4 | 12.9 | 6 | 22.2 | 12 | 38.7 | 3 | 10.7 |
| Irrelevant whether others support | 9 | 7.7 | 3 | 9.7 | 1 | 3.7 | 1 | 3.2 | 4 | 14.3 |
| Care what they look like / fashion | 7 | 6.0 | 1 | 3.2 | 1 | 3.7 | 2 | 6.5 | 3 | 10.7 |
| People who want to be tan / in the sun | 6 | 5.1 | 4 | 12.9 | 1 | 3.7 | 1 | 3.2 | 0 | 0.0 |
| Those not concerned about or understand sun damage | 5 | 4.3 | 2 | 6.5 | 1 | 3.7 | 2 | 6.5 | 0 | 0.0 |
| Sunscreen companies | 4 | 3.4 | 2 | 6.5 | 0 | 0.0 | 1 | 3.2 | 1 | 3.6 |
| Don't know | 4 | 3.4 | 1 | 3.2 | 0 | 0.0 | 2 | 6.5 | 1 | 3.6 |
| People at the beach / pool / lifeguards | 4 | 3.4 | 0 | 0.0 | 0 | 0.0 | 2 | 6.5 | 2 | 7.1 |
| Fitness enthusiasts | 3 | 2.6 | 1 | 3.2 | 1 | 3.7 | 1 | 3.2 | 0 | 0.0 |
| Those who overheat | 3 | 2.6 | 2 | 6.5 | 1 | 3.7 | 0 | 0.0 | 0 | 0.0 |
| Stupid / uneducated people | 3 | 2.6 | 1 | 3.2 | 1 | 3.7 | 0 | 0.0 | 1 | 3.6 |
| Family | 2 | 1.7 | 1 | 3.2 | 0 | 0.0 | 1 | 3.2 | 0 | 0.0 |
| Friends | 2 | 1.7 | 1 | 3.2 | 1 | 3.7 | 0 | 0.0 | 0 | 0.0 |
| People who work outside | 2 | 1.7 | 0 | 0.0 | 1 | 3.7 | 1 | 3.2 | 0 | 0.0 |
| Men | 2 | 1.7 | 0 | 0.0 | 1 | 3.7 | 0 | 0.0 | 1 | 3.6 |
| Rednecks / Trump fans / right wing / Republicans | 2 | 1.7 | 1 | 3.2 | 1 | 3.7 | 0 | 0.0 | 0 | 0.0 |
| People susceptible to peer pressure | 2 | 1.7 | 0 | 0.0 | 0 | 0.0 | 1 | 3.2 | 1 | 3.6 |
| Teammates | 1 | 0.9 | 0 | 0.0 | 1 | 3.7 | 0 | 0.0 | 0 | 0.0 |
| Middle age women | 1 | 0.9 | 1 | 3.2 | 0 | 0.0 | 0 | 0.0 | 0 | 0.0 |
| Swimsuit companies | 1 | 0.9 | 0 | 0.0 | 0 | 0.0 | 1 | 3.2 | 0 | 0.0 |
| Stranger | 1 | 0.9 | 0 | 0.0 | 0 | 0.0 | 1 | 3.2 | 0 | 0.0 |
| People who want to show / see skin | 1 | 0.9 | 0 | 0.0 | 1 | 3.7 | 0 | 0.0 | 0 | 0.0 |
| Darker skin people | 1 | 0.9 | 0 | 0.0 | 0 | 0.0 | 0 | 0.0 | 1 | 3.6 |
| **Likely to Use** |  |  |  |  |  |  |  |  |  |  |
| Older people | 46 | 39.3 | 15 | 48.4 | 7 | 25.9 | 18 | 58.1 | 6 | 21.4 |
| People with personal or family history of skin | 33 | 28.2 | 9 | 29.0 | 3 | 11.1 | 13 | 41.9 | 8 | 28.6 |
| Children | 32 | 27.4 | 15 | 48.4 | 5 | 18.5 | 8 | 25.8 | 4 | 14.3 |
| People with fair skin / prone to sunburn | 30 | 25.6 | 6 | 19.4 | 9 | 33.3 | 5 | 16.1 | 10 | 35.7 |
| People who are active outside (e.g. doing work / workouts) | 19 | 16.2 | 5 | 16.1 | 6 | 22.2 | 5 | 16.1 | 3 | 10.7 |
| People who care about health / sun safety | 11 | 9.4 | 2 | 6.5 | 4 | 14.8 | 4 | 12.9 | 1 | 3.6 |
| Healthcare providers | 8 | 6.8 | 2 | 6.5 | 0 | 0.0 | 5 | 16.1 | 1 | 3.6 |
| Mothers | 4 | 3.4 | 0 | 0.0 | 1 | 3.7 | 2 | 6.5 | 1 | 3.6 |
| People with sensitive skin / allergic to sun | 4 | 3.4 | 1 | 3.2 | 1 | 3.7 | 1 | 3.2 | 1 | 3.6 |
| Smart people | 4 | 3.4 | 0 | 0.0 | 1 | 3.7 | 1 | 3.2 | 2 | 7.1 |
| Scientists / public health people | 3 | 2.6 | 2 | 6.5 | 0 | 0.0 | 1 | 3.2 | 0 | 0.0 |
| Those who take medication that make them sensitive to the sun | 3 | 2.6 | 2 | 6.5 | 1 | 3.7 | 0 | 0.0 | 0 | 0.0 |
| Democrats | 2 | 1.7 | 0 | 0.0 | 1 | 3.7 | 0 | 0.0 | 1 | 3.6 |
| Family | 2 | 1.7 | 1 | 3.2 | 0 | 0.0 | 0 | 0.0 | 1 | 3.6 |
| Those worried about aging | 2 | 1.7 | 1 | 3.2 | 1 | 3.7 | 0 | 0.0 | 0 | 0.0 |
| Middle age adults | 2 | 1.7 | 1 | 3.2 | 0 | 0.0 | 1 | 3.2 | 0 | 0.0 |
| Women | 2 | 1.7 | 0 | 0.0 | 2 | 7.4 | 0 | 0.0 | 0 | 0.0 |
| Political leaders | 1 | 0.9 | 0 | 0.0 | 0 | 0.0 | 1 | 3.2 | 0 | 0.0 |
| People who don't care what others think | 1 | 0.9 | 0 | 0.0 | 0 | 0.0 | 1 | 3.2 | 0 | 0.0 |
| People required to for work (dress code / safety) | 1 | 0.9 | 0 | 0.0 | 0 | 0.0 | 1 | 3.2 | 0 | 0.0 |
| Everyone | 1 | 0.9 | 0 | 0.0 | 1 | 3.7 | 0 | 0.0 | 0 | 0.0 |
| People afraid of sun / people who worry | 1 | 0.9 | 0 | 0.0 | 0 | 0.0 | 0 | 0.0 | 1 | 3.6 |
| People who can afford protective clothing / own protective clothing | 1 | 0.9 | 0 | 0.0 | 0 | 0.0 | 1 | 3.2 | 0 | 0.0 |
| Other | 1 | 0.9 | 0 | 0.0 | 0 | 0.0 | 1 | 3.2 | 0 | 0.0 |
| People conscious about their weight | 1 | 0.9 | 0 | 0.0 | 0 | 0.0 | 0 | 0.0 | 0 | 0.0 |
| **Unlikely to Use** |  |  |  |  |  |  |  |  |  |  |
| Young people | 59 | 50.4 | 18 | 58.1 | 12 | 44.4 | 21 | 67.7 | 8 | 28.6 |
| People who want to be tan / in the sun | 17 | 14.5 | 5 | 16.1 | 4 | 14.8 | 4 | 12.9 | 4 | 14.3 |
| People who are active outside (e.g. doing work/workouts) | 17 | 14.5 | 2 | 6.5 | 8 | 29.6 | 5 | 16.1 | 2 | 7.1 |
| Those who don't think / worry about sun risks | 10 | 8.5 | 4 | 12.9 | 0 | 0.0 | 4 | 12.9 | 2 | 7.1 |
| People with dark skin | 9 | 7.7 | 1 | 3.2 | 4 | 14.8 | 1 | 3.2 | 3 | 10.7 |
| People who don't burn easily or care about | 6 | 5.1 | 1 | 3.2 | 1 | 3.7 | 2 | 6.5 | 2 | 7.1 |
| People who care about fashion / looks | 6 | 5.1 | 3 | 9.7 | 1 | 3.7 | 0 | 0.0 | 2 | 7.1 |
| Not educated / dumb people | 5 | 4.3 | 2 | 6.5 | 0 | 0.0 | 1 | 3.2 | 2 | 7.1 |
| People who can't afford it / do not have protective clothing | 5 | 4.3 | 3 | 9.7 | 1 | 3.7 | 1 | 3.2 | 0 | 0.0 |
| Older people | 5 | 4.3 | 2 | 6.5 | 0 | 0.0 | 1 | 3.2 | 2 | 7.1 |
| Most people / everyone | 4 | 3.4 | 0 | 0.0 | 0 | 0.0 | 3 | 9.7 | 1 | 3.6 |
| People who get hot | 3 | 2.6 | 1 | 3.2 | 1 | 3.7 | 0 | 0.0 | 1 | 3.6 |
| Women | 3 | 2.6 | 1 | 3.2 | 1 | 3.7 | 0 | 0.0 | 1 | 3.6 |
| Me | 2 | 1.7 | 1 | 3.2 | 0 | 0.0 | 1 | 3.2 | 0 | 0.0 |
| People who are overweight | 2 | 1.7 | 1 | 3.2 | 1 | 3.7 | 0 | 0.0 | 0 | 0.0 |
| People are allowed to make their own choices | 2 | 1.7 | 0 | 0.0 | 0 | 0.0 | 0 | 0.0 | 2 | 7.1 |
| Family / friends | 2 | 1.7 | 2 | 6.5 | 0 | 0.0 | 0 | 0.0 | 0 | 0.0 |
| Men | 2 | 1.7 | 0 | 0.0 | 1 | 3.7 | 1 | 3.2 | 0 | 0.0 |
| People who care about what others think | 2 | 1.7 | 1 | 3.2 | 0 | 0.0 | 1 | 3.2 | 0 | 0.0 |
| Republicans | 1 | 0.9 | 0 | 0.0 | 1 | 3.7 | 0 | 0.0 | 0 | 0.0 |
| People who don't want tan lines | 1 | 0.9 | 0 | 0.0 | 1 | 3.7 | 0 | 0.0 | 0 | 0.0 |
| People attending outdoor events | 1 | 0.9 | 0 | 0.0 | 0 | 0.0 | 1 | 3.2 | 0 | 0.0 |
| Contrarians | 1 | 0.9 | 0 | 0.0 | 0 | 0.0 | 0 | 0.0 | 1 | 3.6 |
| People who don't get to be outdoors a lot | 1 | 0.9 | 1 | 3.2 | 0 | 0.0 | 0 | 0.0 | 0 | 0.0 |
| ***Control*** |  |  |  |  |  |  |  |  |  |  |
| **Facilitators / Easier to Use** |  |  |  |  |  |  |  |  |  |  |
| Lightweight / cool / comfortable / loose clothing | 48 | 41.0 | 15 | 48.4 | 12 | 44.4 | 10 | 32.3 | 11 | 39.3 |
| Cool / breezy day / not humid day | 20 | 17.1 | 6 | 19.4 | 4 | 14.8 | 6 | 19.4 | 4 | 14.3 |
| Reasonably priced / financial incentive | 20 | 17.1 | 5 | 16.1 | 2 | 7.4 | 8 | 25.8 | 5 | 17.9 |
| Accessible / own / remembered / have clothes nearby | 18 | 15.4 | 5 | 16.1 | 5 | 18.5 | 3 | 9.7 | 5 | 17.9 |
| Moisture wicking / dry quickly | 8 | 6.8 | 1 | 3.2 | 2 | 7.4 | 3 | 9.7 | 2 | 7.1 |
| Fashionable clothing / more options | 7 | 6.0 | 4 | 12.9 | 1 | 3.7 | 2 | 6.5 | 0 | 0.0 |
| No additional facilitators needed | 5 | 4.3 | 0 | 0.0 | 1 | 3.7 | 2 | 6.5 | 2 | 7.1 |
| Knowing less likely to get skin cancer / already had skin cancer | 4 | 3.4 | 2 | 6.5 | 1 | 3.7 | 1 | 3.2 | 0 | 0.0 |
| Not being physically active | 4 | 3.4 | 1 | 3.2 | 2 | 7.4 | 0 | 0.0 | 1 | 3.6 |
| Shade available | 3 | 2.6 | 1 | 3.2 | 0 | 0.0 | 2 | 6.5 | 0 | 0.0 |
| Design features (zippers / pockets / fast drying / waterproof) | 3 | 2.6 | 1 | 3.2 | 1 | 3.7 | 1 | 3.2 | 0 | 0.0 |
| Access to water | 3 | 2.6 | 2 | 6.5 | 0 | 0.0 | 1 | 3.2 | 0 | 0.0 |
| Doing water sports | 2 | 1.7 | 0 | 0.0 | 1 | 3.7 | 1 | 3.2 | 0 | 0.0 |
| Easy to take off / not restrictive | 2 | 1.7 | 0 | 0.0 | 1 | 3.7 | 0 | 0.0 | 1 | 3.6 |
| Sensitive skin | 2 | 1.7 | 2 | 6.5 | 0 | 0.0 | 0 | 0.0 | 0 | 0.0 |
| Planning to be outside for a long time | 2 | 1.7 | 0 | 0.0 | 0 | 0.0 | 1 | 3.2 | 1 | 3.6 |
| Available department stores / all stores | 2 | 1.7 | 1 | 3.2 | 0 | 0.0 | 1 | 3.2 | 0 | 0.0 |
| Somewhere you don't know a lot of people / pay less attention to what people say / few people | 2 | 1.7 | 0 | 0.0 | 1 | 3.7 | 1 | 3.2 | 0 | 0.0 |
| Social acceptance | 2 | 1.7 | 2 | 6.5 | 0 | 0.0 | 0 | 0.0 | 0 | 0.0 |
| Sunscreen not available | 2 | 1.7 | 2 | 6.5 | 0 | 0.0 | 0 | 0.0 | 0 | 0.0 |
| Being sunburned | 2 | 1.7 | 0 | 0.0 | 2 | 7.4 | 0 | 0.0 | 0 | 0.0 |
| Doctor told me to wear | 1 | 0.9 | 1 | 3.2 | 0 | 0.0 | 0 | 0.0 | 0 | 0.0 |
| No shade nearby | 1 | 0.9 | 0 | 0.0 | 1 | 3.7 | 0 | 0.0 | 0 | 0.0 |
| Allows vitamin D while restricting UV | 1 | 0.9 | 0 | 0.0 | 1 | 3.7 | 0 | 0.0 | 0 | 0.0 |
| Hide skin | 1 | 0.9 | 0 | 0.0 | 0 | 0.0 | 1 | 3.2 | 0 | 0.0 |
| Working outside | 1 | 0.9 | 0 | 0.0 | 0 | 0.0 | 0 | 0.0 | 1 | 3.6 |
| Older age | 1 | 0.9 | 0 | 0.0 | 1 | 3.7 | 0 | 0.0 | 0 | 0.0 |
| Bugs | 1 | 0.9 | 0 | 0.0 | 1 | 3.7 | 0 | 0.0 | 0 | 0.0 |
| Access to restrooms | 1 | 0.9 | 1 | 3.2 | 0 | 0.0 | 0 | 0.0 | 0 | 0.0 |
| **Barriers / Harder to Use** |  |  |  |  |  |  |  |  |  |  |
| Hot / muggy | 59 | 50.4 | 18 | 58.1 | 14 | 51.9 | 15 | 48.4 | 12 | 42.9 |
| When active | 26 | 22.2 | 8 | 25.8 | 9 | 33.3 | 2 | 6.5 | 7 | 25.0 |
| Cost | 21 | 17.9 | 6 | 19.4 | 1 | 3.7 | 7 | 22.6 | 7 | 25.0 |
| Not accessible / don't own | 15 | 12.8 | 4 | 12.9 | 2 | 7.4 | 5 | 16.1 | 4 | 14.3 |
| Not comfortable | 7 | 6.0 | 1 | 3.2 | 0 | 0.0 | 4 | 12.9 | 2 | 7.1 |
| No barriers | 5 | 4.3 | 0 | 0.0 | 2 | 7.4 | 1 | 3.2 | 2 | 7.1 |
| Clothing heavy | 5 | 4.3 | 1 | 3.2 | 0 | 0.0 | 1 | 3.2 | 3 | 10.7 |
| Can't feel warmth / sun on skin | 5 | 4.3 | 2 | 6.5 | 3 | 11.1 | 0 | 0.0 | 0 | 0.0 |
| Sweating | 5 | 4.3 | 0 | 0.0 | 3 | 11.1 | 2 | 6.5 | 0 | 0.0 |
| Not fashionable | 4 | 3.4 | 1 | 3.2 | 0 | 0.0 | 1 | 3.2 | 2 | 7.1 |
| Too tight / Restrictive | 4 | 3.4 | 1 | 3.2 | 0 | 0.0 | 1 | 3.2 | 2 | 7.1 |
| Other people not wearing protective clothing / perceptions / other people's comments | 4 | 3.4 | 0 | 0.0 | 0 | 0.0 | 1 | 3.2 | 0 | 0.0 |
| Other | 4 | 3.4 | 2 | 6.5 | 0 | 0.0 | 1 | 3.2 | 0 | 0.0 |
| Forget / don't plan ahead | 2 | 1.7 | 0 | 0.0 | 1 | 3.7 | 1 | 3.2 | 0 | 0.0 |
| No breeze / wind | 2 | 1.7 | 0 | 0.0 | 0 | 0.0 | 1 | 3.2 | 1 | 3.6 |
| Clothing not durable for outdoor work / farming | 2 | 1.7 | 0 | 0.0 | 0 | 0.0 | 1 | 3.2 | 1 | 3.6 |
| No access to shade / indoor area | 2 | 1.7 | 0 | 0.0 | 1 | 3.7 | 1 | 3.2 | 0 | 0.0 |
| Easy to find / purchase / unavailable in store | 1 | 0.9 | 0 | 0.0 | 0 | 0.0 | 1 | 3.2 | 0 | 0.0 |
| Already sunburnt | 1 | 0.9 | 0 | 0.0 | 0 | 0.0 | 1 | 3.2 | 0 | 0.0 |
| Lack of water to drink | 1 | 0.9 | 0 | 0.0 | 0 | 0.0 | 1 | 3.2 | 0 | 0.0 |
| Tan lines | 1 | 0.9 | 1 | 3.2 | 0 | 0.0 | 0 | 0.0 | 0 | 0.0 |
| Hide skin | 1 | 0.9 | 0 | 0.0 | 0 | 0.0 | 1 | 3.2 | 0 | 0.0 |
| Have sunscreen available | 1 | 0.9 | 1 | 3.2 | 0 | 0.0 | 0 | 0.0 | 0 | 0.0 |
| Perception / other people's comments | 1 | 0.9 | 1 | 3.2 | 1 | 3.7 | 2 | 6.5 | 0 | 0.0 |
| No restroom nearby | 1 | 0.9 | 1 | 3.2 | 0 | 0.0 | 0 | 0.0 | 0 | 0.0 |
| Ego | 1 | 0.9 | 0 | 0.0 | 1 | 3.7 | 0 | 0.0 | 0 | 0.0 |
