# Supplementary material for: Sun protection behavior beliefs among adults living in rural United States: A qualitative study in Minnesota
Source: PLoS One. 2025 Sep 12;20(9):e0331685. doi: 10.1371/journal.pone.0331685 (PMC12431656; doi:10.1371/journal.pone.0331685)
Supplement: S4 Table — (DOCX) [file pone.0331685.s008.docx]

**S4 Table. All beliefs about wearing a hat to reduce sun exposure and prevent sunburn on typical sunny day in the summer.**

|  | **Overall**  **N=105** | | **18-39 Female**  **N=31** | | **18-39 Male**  **N=20** | | **40-60 Female**  **N=28** | | **40-60 Male**  **N=26** | |
| --- | --- | --- | --- | --- | --- | --- | --- | --- | --- | --- |
| **Beliefs** | **n** | **%** | **n** | **%** | **n** | **%** | **n** | **%** | **n** | **%** |
| ***Outcome*** |  |  |  |  |  |  |  |  |  |  |
| **Good/Positive** |  |  |  |  |  |  |  |  |  |  |
| Less cancer / exposure / sunburn | 98 | 93.3 | 30 | 96.8 | 18 | 90.0 | 25 | 89.3 | 25 | 96.2 |
| Sun out of eyes | 24 | 22.9 | 9 | 29.0 | 7 | 35.0 | 5 | 17.9 | 3 | 11.5 |
| Protection from heat / stay cool | 22 | 21.0 | 8 | 25.8 | 4 | 20.0 | 7 | 25.0 | 3 | 11.5 |
| Better than sunscreen / avoid sunscreen | 9 | 8.6 | 5 | 16.1 | 2 | 10.0 | 2 | 7.1 | 0 | 0.0 |
| Prevent aging / less skin damage | 9 | 8.6 | 4 | 12.9 | 0 | 0.0 | 5 | 17.9 | 0 | 0.0 |
| Looks good / stylish | 8 | 7.6 | 0 | 0.0 | 2 | 10.0 | 5 | 17.9 | 1 | 3.9 |
| Stay outside longer | 4 | 3.8 | 2 | 6.5 | 1 | 5.0 | 0 | 0.0 | 1 | 3.9 |
| Healthy lifestyle | 2 | 1.9 | 0 | 0.0 | 0 | 0.0 | 1 | 3.6 | 1 | 3.9 |
| Less sweat on face / in eyes | 2 | 1.9 | 1 | 3.2 | 1 | 5.0 | 0 | 0.0 | 0 | 0.0 |
| Protect hair / don't have to style hair | 2 | 1.9 | 0 | 0.0 | 0 | 0.0 | 2 | 7.1 | 0 | 0.0 |
| Keep bugs off head | 1 | 1.0 | 0 | 0.0 | 1 | 5.0 | 0 | 0.0 | 0 | 0.0 |
| Rain | 1 | 1.0 | 0 | 0.0 | 1 | 5.0 | 0 | 0.0 | 0 | 0.0 |
| **Bad/Negative** |  |  |  |  |  |  |  |  |  |  |
| Blows off / easy to lose | 48 | 45.7 | 20 | 64.5 | 8 | 40.0 | 12 | 42.9 | 8 | 30.8 |
| Head gets hot / sweaty | 38 | 36.2 | 12 | 38.7 | 7 | 35.0 | 14 | 50.0 | 5 | 19.2 |
| Inconvenient / interferes with work/task | 29 | 27.6 | 12 | 38.7 | 8 | 40.0 | 7 | 25.0 | 2 | 7.7 |
| Not fashionable / does not look good / difficult to find one they like | 25 | 23.8 | 6 | 19.4 | 5 | 25.0 | 7 | 25.0 | 7 | 26.9 |
| Uncomfortable / does not fit well | 19 | 18.1 | 5 | 16.1 | 6 | 30.0 | 5 | 17.9 | 3 | 11.5 |
| Nothing negative about wearing a hat | 16 | 15.2 | 3 | 9.7 | 4 | 20.0 | 2 | 7.1 | 7 | 26.9 |
| Hair not styled properly / messes up hair | 12 | 11.4 | 3 | 9.7 | 1 | 5.0 | 7 | 25.0 | 1 | 3.9 |
| Obstructed view / cannot see much | 10 | 9.5 | 1 | 3.2 | 3 | 15.0 | 4 | 14.3 | 2 | 7.7 |
| Don't like hats / not my thing | 8 | 7.6 | 1 | 3.2 | 1 | 5.0 | 3 | 10.7 | 3 | 11.5 |
| Forget to bring hat / need to remember / transport | 8 | 7.6 | 5 | 16.1 | 0 | 0.0 | 2 | 7.1 | 1 | 3.9 |
| Other's judgement / looking / may get teased | 8 | 7.6 | 3 | 9.7 | 2 | 10.0 | 2 | 7.1 | 1 | 3.9 |
| Don't own any | 5 | 4.8 | 1 | 3.2 | 1 | 5.0 | 2 | 7.1 | 1 | 3.9 |
| Uneven tan / no tan on face | 4 | 3.8 | 1 | 3.2 | 1 | 5.0 | 1 | 3.6 | 1 | 3.9 |
| Cost | 3 | 2.9 | 0 | 0.0 | 0 | 0.0 | 2 | 7.1 | 1 | 3.9 |
| Forget to put on sunscreen | 3 | 2.9 | 1 | 3.2 | 1 | 5.0 | 1 | 3.6 | 0 | 0.0 |
| Leaves mark on forehead | 2 | 1.9 | 2 | 6.5 | 0 | 0.0 | 0 | 0.0 | 0 | 0.0 |
| Shade | 2 | 1.9 | 0 | 0.0 | 0 | 0.0 | 1 | 3.6 | 1 | 3.9 |
| Can't wear in the water | 1 | 1.0 | 0 | 0.0 | 1 | 5.0 | 0 | 0.0 | 0 | 0.0 |
| ***Normative*** |  |  |  |  |  |  |  |  |  |  |
| **Approve/Support Use** |  |  |  |  |  |  |  |  |  |  |
| Older people | 26 | 24.8 | 11 | 35.5 | 5 | 25.0 | 5 | 17.9 | 5 | 19.2 |
| Most people / everyone | 24 | 22.9 | 7 | 22.6 | 6 | 30.0 | 8 | 28.6 | 3 | 11.5 |
| Family / friends | 23 | 21.9 | 5 | 16.2 | 4 | 20.0 | 10 | 35.8 | 4 | 15.4 |
| Does not matter / irrelevant whether someone approves | 12 | 11.4 | 2 | 6.5 | 2 | 10.0 | 4 | 14.3 | 4 | 15.4 |
| Those who are active outside (e.g. doing work / workouts) | 10 | 9.5 | 2 | 6.5 | 7 | 35.0 | 1 | 3.6 | 0 | 0.0 |
| Those with knowledge about effects of sun exposure / people concerned about health | 7 | 6.7 | 2 | 6.5 | 0 | 0.0 | 2 | 7.1 | 3 | 11.5 |
| Healthcare providers | 7 | 6.7 | 0 | 0.0 | 1 | 5.0 | 3 | 10.7 | 3 | 11.5 |
| People who like hats / who (stereotypically) wear hats anyway | 6 | 5.7 | 1 | 3.2 | 3 | 15.0 | 0 | 0.0 | 2 | 7.7 |
| Parents/people with children | 5 | 4.8 | 3 | 9.7 | 1 | 5.0 | 1 | 3.6 | 0 | 0.0 |
| Women | 4 | 3.8 | 2 | 6.5 | 1 | 5.0 | 1 | 3.6 | 0 | 0.0 |
| Beach goers / fishermen | 3 | 2.9 | 1 | 3.2 | 1 | 5.0 | 1 | 3.6 | 0 | 0.0 |
| I don't know | 3 | 2.9 | 1 | 3.2 | 0 | 0.0 | 1 | 3.6 | 1 | 3.9 |
| Natural-minded people / hippies / naturalists | 2 | 1.9 | 1 | 3.2 | 0 | 0.0 | 1 | 3.6 | 0 | 0.0 |
| People who have pale skin / sunburn easily | 2 | 1.9 | 1 | 3.2 | 1 | 5.0 | 0 | 0.0 | 0 | 0.0 |
| People with personal or family history of skin cancer | 2 | 1.9 | 1 | 3.2 | 0 | 0.0 | 0 | 0.0 | 1 | 3.9 |
| Goths | 1 | 1.0 | 1 | 3.2 | 0 | 0.0 | 0 | 0.0 | 0 | 0.0 |
| People less physically active | 1 | 1.0 | 1 | 3.2 | 0 | 0.0 | 0 | 0.0 | 0 | 0.0 |
| People who sell hats | 1 | 1.0 | 0 | 0.0 | 0 | 0.0 | 1 | 3.6 | 0 | 0.0 |
| Employers / coworkers | 1 | 1.0 | 0 | 0.0 | 0 | 0.0 | 0 | 0.0 | 1 | 3.9 |
| People with less hair | 1 | 1.0 | 0 | 0.0 | 0 | 0.0 | 1 | 3.6 | 0 | 0.0 |
| People who wear sunscreen | 1 | 1.0 | 1 | 3.2 | 0 | 0.0 | 0 | 0.0 | 0 | 0.0 |
| **Disapprove/Not Support Use** |  |  |  |  |  |  |  |  |  |  |
| No one disapproves | 34 | 32.4 | 10 | 32.3 | 6 | 30.0 | 9 | 32.1 | 9 | 34.6 |
| Young people | 25 | 23.8 | 9 | 29.0 | 6 | 30.0 | 5 | 17.9 | 5 | 19.2 |
| Does not matter / irrelevant whether someone approves | 14 | 13.3 | 3 | 9.7 | 1 | 5.0 | 5 | 17.9 | 5 | 19.2 |
| People who care about being fashionable / stylish / superficial / think wearing hats is goofy or weird / judgmental | 9 | 8.6 | 2 | 6.5 | 4 | 20.0 | 1 | 3.6 | 2 | 7.7 |
| I don't know | 7 | 6.7 | 4 | 12.9 | 1 | 5.0 | 0 | 0.0 | 2 | 7.7 |
| Those physically active / cycling / swimming / working outside | 4 | 3.8 | 2 | 6.5 | 2 | 10.0 | 0 | 0.0 | 0 | 0.0 |
| Strangers | 4 | 3.8 | 1 | 3.2 | 1 | 5.0 | 1 | 3.6 | 1 | 3.9 |
| Specific family members | 4 | 3.8 | 0 | 0.0 | 0 | 0.0 | 4 | 14.3 | 0 | 0.0 |
| Friends | 3 | 2.9 | 1 | 3.2 | 2 | 10.0 | 0 | 0.0 | 0 | 0.0 |
| City people | 1 | 1.0 | 0 | 0.0 | 0 | 0.0 | 0 | 0.0 | 1 | 3.9 |
| Those who do not worry about sun exposure | 1 | 1.0 | 0 | 0.0 | 1 | 5.0 | 0 | 0.0 | 0 | 0.0 |
| People when it is crowded | 1 | 1.0 | 0 | 0.0 | 0 | 0.0 | 0 | 0.0 | 1 | 3.9 |
| Most | 1 | 1.0 | 0 | 0.0 | 0 | 0.0 | 1 | 3.6 | 0 | 0.0 |
| **Likely to Use** |  |  |  |  |  |  |  |  |  |  |
| Older people | 51 | 48.6 | 20 | 64.5 | 11 | 55.0 | 9 | 32.1 | 11 | 42.3 |
| People who are active outside (e.g. doing work / workouts) | 32 | 30.5 | 8 | 25.8 | 12 | 60.0 | 7 | 25.0 | 5 | 19.2 |
| Women | 16 | 15.2 | 5 | 16.1 | 4 | 20.0 | 5 | 17.9 | 2 | 7.7 |
| Those with knowledge about effects of sun exposure / people concerned about health | 9 | 8.6 | 3 | 9.7 | 0 | 0.0 | 5 | 17.9 | 1 | 3.9 |
| People who have fair skin / red hair / sunburn easily | 9 | 8.6 | 1 | 3.2 | 2 | 10.0 | 2 | 7.1 | 4 | 15.4 |
| People with personal or family history of skin cancer | 6 | 5.7 | 2 | 6.5 | 1 | 5.0 | 1 | 3.6 | 2 | 7.7 |
| Children | 6 | 5.7 | 2 | 6.5 | 1 | 5.0 | 2 | 7.1 | 1 | 3.9 |
| People who like hats / who (stereotypically) wear hats anyway | 4 | 3.8 | 0 | 0.0 | 1 | 5.0 | 2 | 7.1 | 1 | 3.9 |
| Healthcare providers / scientists | 3 | 2.9 | 0 | 0.0 | 0 | 0.0 | 3 | 10.7 | 0 | 0.0 |
| Don't are about being cool / fashionable | 2 | 1.9 | 1 | 3.2 | 1 | 5.0 | 0 | 0.0 | 0 | 0.0 |
| I don't know | 2 | 1.9 | 1 | 3.2 | 0 | 0.0 | 0 | 0.0 | 1 | 3.9 |
| People sensitive to light / sun | 2 | 1.9 | 0 | 0.0 | 0 | 0.0 | 2 | 7.1 | 0 | 0.0 |
| Men | 1 | 1.0 | 0 | 0.0 | 0 | 0.0 | 1 | 3.6 | 0 | 0.0 |
| Goths | 1 | 1.0 | 1 | 3.2 | 0 | 0.0 | 0 | 0.0 | 0 | 0.0 |
| People less physically active | 1 | 1.0 | 1 | 3.2 | 0 | 0.0 | 0 | 0.0 | 0 | 0.0 |
| Natural-minded people / hippies / naturalists | 1 | 1.0 | 0 | 0.0 | 0 | 0.0 | 1 | 3.6 | 0 | 0.0 |
| Live in warm areas | 1 | 1.0 | 0 | 0.0 | 0 | 0.0 | 0 | 0.0 | 1 | 3.9 |
| People with less/short hair | 1 | 1.0 | 0 | 0.0 | 0 | 0.0 | 1 | 3.6 | 0 | 0.0 |
| People who plan ahead / organized people | 1 | 1.0 | 1 | 3.2 | 0 | 0.0 | 0 | 0.0 | 0 | 0.0 |
| Outgoing people | 1 | 1.0 | 0 | 0.0 | 1 | 5.0 | 0 | 0.0 | 0 | 0.0 |
| Parents/people with children | 1 | 1.0 | 0 | 0.0 | 1 | 5.0 | 0 | 0.0 | 0 | 0.0 |
| **Unlikely to Use** |  |  |  |  |  |  |  |  |  |  |
| Young people | 52 | 49.5 | 18 | 58.1 | 12 | 60.0 | 13 | 46.4 | 9 | 34.6 |
| People who are active outside (e.g. doing work / workouts) | 15 | 14.3 | 3 | 9.7 | 3 | 15.0 | 3 | 10.7 | 6 | 23.1 |
| Men | 13 | 12.4 | 5 | 16.1 | 1 | 5.0 | 5 | 17.9 | 2 | 7.7 |
| People who don't like hats / find hats uncomfortable / style-conscious people | 11 | 10.5 | 2 | 6.5 | 2 | 10.0 | 6 | 21.4 | 1 | 3.9 |
| Those who do not care/worry about sun exposure and risks | 5 | 4.8 | 0 | 0.0 | 1 | 5.0 | 3 | 10.7 | 1 | 3.9 |
| Most people / everyone | 5 | 4.8 | 2 | 6.5 | 1 | 5.0 | 0 | 0.0 | 2 | 7.7 |
| People working or spending most of their time indoors | 4 | 3.8 | 1 | 3.2 | 2 | 10.0 | 0 | 0.0 | 1 | 3.9 |
| People with dark skin | 2 | 1.9 | 0 | 0.0 | 0 | 0.0 | 0 | 0.0 | 2 | 7.7 |
| People who use other sun protective measures (e.g. sunscreen) | 2 | 1.9 | 0 | 0.0 | 1 | 5.0 | 1 | 3.6 | 0 | 0.0 |
| No one is unlikely to use | 2 | 1.9 | 1 | 3.2 | 0 | 0.0 | 0 | 0.0 | 1 | 3.9 |
| People who did not grow up with sun protective measures | 2 | 1.9 | 0 | 0.0 | 0 | 0.0 | 0 | 0.0 | 0 | 0.0 |
| Urban people | 2 | 1.9 | 0 | 0.0 | 1 | 5.0 | 0 | 0.0 | 1 | 3.9 |
| People with long hair | 1 | 1.0 | 0 | 0.0 | 0 | 0.0 | 1 | 3.6 | 0 | 0.0 |
| Too lazy to put a hat on | 1 | 1.0 | 0 | 0.0 | 0 | 0.0 | 1 | 3.6 | 0 | 0.0 |
| Specific family members | 1 | 1.0 | 0 | 0.0 | 0 | 0.0 | 1 | 3.6 | 0 | 0.0 |
| People who want to be tan / in the sun | 1 | 1.0 | 0 | 0.0 | 0 | 0.0 | 1 | 3.6 | 0 | 0.0 |
| Forget to wear hat | 1 | 1.0 | 1 | 3.2 | 0 | 0.0 | 0 | 0.0 | 0 | 0.0 |
| Don't have enough money for multiple hats | 1 | 1.0 | 0 | 0.0 | 1 | 5.0 | 0 | 0.0 | 0 | 0.0 |
| People who have not had experience with skin cancer | 1 | 1.0 | 0 | 0.0 | 0 | 0.0 | 0 | 0.0 | 1 | 3.9 |
| I don't know | 1 | 1.0 | 1 | 3.2 | 0 | 0.0 | 0 | 0.0 | 1 | 3.9 |
| People who live in cool states | 1 | 1.0 | 0 | 0.0 | 0 | 0.0 | 0 | 0.0 | 1 | 3.9 |
| Women | 1 | 1.0 | 0 | 0.0 | 0 | 0.0 | 1 | 3.6 | 0 | 0.0 |
| ***Control*** |  |  |  |  |  |  |  |  |  |  |
| **Facilitators / Easier to Use** |  |  |  |  |  |  |  |  |  |  |
| Properly fitting / lighter / comfortable / breathable | 27 | 25.7 | 8 | 25.8 | 6 | 30.0 | 12 | 42.9 | 1 | 3.9 |
| Stylish options / more options | 16 | 15.2 | 3 | 9.7 | 4 | 20.0 | 7 | 25.0 | 2 | 7.7 |
| Stay on head | 16 | 15.2 | 7 | 22.6 | 3 | 15.0 | 4 | 14.3 | 2 | 7.7 |
| Own/bought wide brimmed hat(s) | 13 | 12.4 | 6 | 19.4 | 4 | 20.0 | 1 | 3.6 | 2 | 7.7 |
| Not windy | 9 | 8.6 | 5 | 16.1 | 2 | 10.0 | 2 | 7.1 | 0 | 0.0 |
| No additional facilitators needed | 9 | 8.6 | 1 | 3.2 | 1 | 5.0 | 1 | 3.6 | 6 | 23.1 |
| Not working / not active / not doing activity where hat would fall off | 6 | 5.7 | 1 | 3.2 | 3 | 15.0 | 1 | 3.6 | 1 | 3.9 |
| Outside a lot / too much sun exposure / sunny | 5 | 4.8 | 0 | 0.0 | 1 | 5.0 | 2 | 7.1 | 2 | 7.7 |
| Having it easily available / nearby | 4 | 3.8 | 2 | 6.5 | 1 | 5.0 | 1 | 3.6 | 0 | 0.0 |
| Cost reasonable | 3 | 2.9 | 0 | 0.0 | 0 | 0.0 | 2 | 7.1 | 1 | 3.9 |
| Everyone wearing one / socially acceptable | 3 | 2.9 | 0 | 0.0 | 0 | 0.0 | 1 | 3.6 | 2 | 7.7 |
| Hair down / don't care about hair style | 3 | 2.9 | 1 | 3.2 | 0 | 0.0 | 2 | 7.1 | 0 | 0.0 |
| Remember to wear | 2 | 1.9 | 0 | 0.0 | 1 | 5.0 | 1 | 3.6 | 0 | 0.0 |
| Foldable / easy to carry | 2 | 1.9 | 0 | 0.0 | 0 | 0.0 | 2 | 7.1 | 0 | 0.0 |
| Didn't have sunglasses | 2 | 1.9 | 1 | 3.2 | 0 | 0.0 | 1 | 3.6 | 0 | 0.0 |
| At beach / on lake | 2 | 1.9 | 1 | 3.2 | 0 | 0.0 | 0 | 0.0 | 1 | 3.9 |
| I don't know | 2 | 1.9 | 0 | 0.0 | 0 | 0.0 | 1 | 3.6 | 1 | 3.9 |
| Not too hot | 2 | 1.9 | 0 | 0.0 | 0 | 0.0 | 0 | 0.0 | 2 | 7.7 |
| Already had sunburn | 1 | 1.0 | 0 | 0.0 | 0 | 0.0 | 0 | 0.0 | 1 | 3.9 |
| Doctor advised | 1 | 1.0 | 0 | 0.0 | 0 | 0.0 | 0 | 0.0 | 1 | 3.9 |
| Previous skin cancer diagnosis | 1 | 1.0 | 0 | 0.0 | 0 | 0.0 | 1 | 3.6 | 0 | 0.0 |
| Being at home | 1 | 1.0 | 1 | 3.2 | 0 | 0.0 | 0 | 0.0 | 0 | 0.0 |
| If outside with family | 1 | 1.0 | 0 | 0.0 | 0 | 0.0 | 1 | 3.6 | 0 | 0.0 |
| **Barriers / Harder to Use** |  |  |  |  |  |  |  |  |  |  |
| Depends on wind / weather | 40 | 38.1 | 11 | 35.5 | 9 | 45.0 | 12 | 42.9 | 8 | 30.8 |
| Doing physical activity / working outside / depends on activity | 21 | 20.0 | 8 | 25.8 | 4 | 20.0 | 5 | 17.9 | 4 | 15.4 |
| Forgot to bring or put on | 14 | 13.3 | 5 | 16.1 | 1 | 5.0 | 6 | 21.4 | 2 | 7.7 |
| Don't own one | 12 | 11.4 | 2 | 6.5 | 3 | 15.0 | 6 | 21.4 | 1 | 3.9 |
| Too hot / sweaty | 12 | 11.4 | 3 | 9.7 | 3 | 15.0 | 3 | 10.7 | 3 | 11.5 |
| Does not look good / fashionable / bulky / difficult to find a hat one likes or fits | 9 | 8.6 | 3 | 9.7 | 2 | 10.0 | 2 | 7.1 | 2 | 7.7 |
| No barriers | 8 | 7.6 | 2 | 6.5 | 2 | 10.0 | 1 | 3.6 | 3 | 11.5 |
| I never wear / don't like | 6 | 5.7 | 1 | 3.2 | 1 | 5.0 | 1 | 3.6 | 3 | 11.5 |
| Hairstyle | 4 | 3.8 | 1 | 3.2 | 1 | 5.0 | 2 | 7.1 | 0 | 0.0 |
| No one else wearing hats | 3 | 2.9 | 1 | 3.2 | 1 | 5.0 | 0 | 0.0 | 1 | 3.9 |
| Crowded / no space | 3 | 2.9 | 3 | 9.7 | 0 | 0.0 | 0 | 0.0 | 0 | 0.0 |
| Inconvenient / nuisance | 3 | 1.0 | 1 | 3.2 | 2 | 10.0 | 0 | 0.0 | 0 | 0.0 |
| Uncomfortable | 2 | 1.9 | 0 | 0.0 | 2 | 10.0 | 0 | 0.0 | 0 | 0.0 |
| Tan lines | 1 | 1.0 | 0 | 0.0 | 0 | 0.0 | 1 | 3.6 | 0 | 0.0 |
| It is easy / already do this | 1 | 1.0 | 0 | 0.0 | 0 | 0.0 | 0 | 0.0 | 1 | 3.9 |
| Not concerned about sun exposure | 1 | 1.0 | 0 | 0.0 | 1 | 5.0 | 0 | 0.0 | 0 | 0.0 |
| Dog will try and grab | 1 | 1.0 | 0 | 0.0 | 0 | 0.0 | 1 | 3.6 | 0 | 0.0 |
